# Population-level spatial patterns of body-surface temperature in older adults: a multicenter infrared thermography study across diverse environmental settings

**DOI:** 10.3389/fphys.2026.1870629

**Published:** 2026-10-28

**Authors:** Siying Dong, Mengchen Tu, Wenzheng Tang, Wenle Li, Zhuqing Li, Yuyang Cai, Wei Wei, Gengshuo Miao, Minghua Bai, Ji Wang, Qi Wang

**Affiliations:** 1 School of Traditional Chinese Medicine, Anhui University of Traditional Chinese Medicine, Hefei, China; 2 National Institute of TCM Constitution and Preventive Medicine, Beijing University of Chinese Medicine, Beijing, China; 3 State Key Lab of Polymer Materials and Engineering, College of Polymer Science and Engineering, Sichuan University, Chengdu, China; 4 School of Traditional Chinese Medicine, Beijing University of Chinese Medicine, Beijing, China; 5 School of Traditional Chinese Medicine, Nanjing University of Chinese Medicine, Nanjing, China

**Keywords:** body-surface temperature, environmental variability, infrared thermography, non-invasive measurement, older adults, population health, spatial patterns

## Abstract

**Background:**

Body-surface temperature reflects peripheral physiological regulation and may provide a useful phenotype for population studies of ageing. Infrared thermography (IRT) enables non-invasive assessment of temperature distribution, but large multicenter evidence remains limited. We aimed to characterise body-surface temperature patterns in older adults and examine their associations with individual and contextual factors.

**Methods:**

In this multicenter observational study, 5,225 adults aged ≥60 years underwent standardized IRT across multiple regions in China. Temperature was measured at 12 predefined trunk regions. Both absolute regional temperature and plane-specific relative temperature (ΔT) were analyzed. Multivariable regression models adjusted for demographic, anthropometric, physiological, and center-related variables were used to assess constitution-by-region interactions, followed by false discovery rate-adjusted region-specific analysis. Sensitivity analysis included absolute-temperature models and leave-one-site-out validation.

**Results:**

A total of 62,700 region-level observations were analyzed. Regional setting was the largest contributor to thermal variability, followed by body composition, whereas constitution explained a relatively small proportion of overall variance (2.0–10.9%). Significant constitution-by-region interactions indicated spatially heterogeneous temperature patterns (χ²=732.41, df=88, p<0.001). Constitution-related differences were localized mainly to abdominal, posterior lumbar, and selected thoracic regions, with modest effect sizes (0.08–0.27 °C) that remained directionally consistent across sensitivity analysis.

**Conclusion:**

Body-surface temperature in older adults exhibits modest but spatially organized variation influenced predominantly by regional setting and body composition. Constitution-related differences are localized and reproducible but should be interpreted as exploratory surface-temperature phenotypes rather than clinically actionable findings.

## 1 Background

As the prevalence of multimorbidity and chronic conditions, including cardiovascular disease, diabetes, and cancer, rises markedly, diagnosis in older adults is frequently confounded by non-specific presentations, such as fatigue or functional decline, rather than classic symptomatology (Head et al., 2021; Bray et al., 2024; Vollset et al., 2024). Current diagnostic frameworks, largely derived from single-disease trials where older adults are under-represented, often lead to compromised diagnostic sensitivity in this population (Haertter et al., 2025; Vart, 2025). Given the ubiquity of multimorbidity, a systematic integrative framework is urgently required to address the complexity of geriatric care (Salisbury et al., 2018). Physiological ageing is characterized by blunted peripheral vascular reactivity and reduced skin perfusion, increasing susceptibility to thermal dysregulation (Kenney and Munce, 2003). Body-surface temperature serves as an accessible biomarker of ageing, potentially reflecting age-related changes in peripheral temperature patterns (Balmain et al., 2018). Dysfunction in thermoregulation is closely linked to frailty, cardiovascular instability, and the risk of multiple chronic conditions (Kenny et al., 2010). Extending beyond a simple physical parameter, body-surface temperature offers a sensitive index of peripheral perfusion, metabolic activity, and autonomic integrity (Nilsson, 1987; Wang et al., 2022). Age-related facial thermal patterns correlate with metabolic and sleep indicators, suggesting thermal features may serve as objective markers of ageing trajectories (Yu et al., 2024). With advances in infrared thermography (IRT), temperature assessment has become rapid and non-invasive, enabling simultaneous capture of temperature gradients, lateral asymmetry, and focal abnormalities across multiple anatomical sites (Kesztyüs et al., 2022; Liu et al., 2025). Although IRT is established in infection screening and circulatory assessment, large-scale multicenter investigations of IRT-derived phenotypes in older populations remain scarce, implying that its potential role in characterizing population-level thermal phenotypes has not yet been fully explored.

Traditional Chinese Medicine (TCM) diagnoses holistic health through four diagnostic pillars (inspection, auscultation/olfaction, inquiry, and palpation). TCM constitution theory, informed by epidemiological evidence, stratifies populations into a Balanced constitution and eight biased constitution types, reflecting stable traits in morphology, physiological function, and psychological state (Li et al., 2021). Previous studies have suggested that constitution type may be associated with differences in gene expression related to physiological regulation, although the underlying biological relevance remains incompletely understood (Li et al., 2017). The TCM constitution was assessed using the validated “Constitution in Chinese Medicine Questionnaire (Elderly Edition)”, which has demonstrated good reliability and validity in prior studies, including internal consistency and test-retest reliability (Song et al., 2022). By profiling overall constitutional status and revealing subtle links to potential pathology, this system offers a framework aligned with the imperatives of chronic disease management in older adults. The questionnaire has been validated as a standardized research instrument and constitution is generally considered a relatively stable individual characteristic, although its biological basis and cross-cultural generalizability remain under investigation.

From an immunometabolic perspective, TCM cold and heat patterns have been proposed to reflect systemic regulatory modes rather than isolated symptoms (Fernández-Cuevas et al., 2015). This aligns with the stable individual traits emphasized in constitution theory, suggesting that cold–heat manifestations vary by constitution type. For instance, reduced expression of thyroid hormone receptor β in Yang-deficiency constitution may impair thermogenesis, contributing to cold intolerance (Ludwig et al., 2014). These observations imply that infrared-derived body-surface temperature may exhibit a measurable correspondence with constitution type. However, despite the conceptual links between constitution theory and thermoregulation-related observations, high-quality evidence from large-scale multicenter standardized studies remains limited. Infrared body-surface temperature may provide an exploratory quantitative phenotype for examining the relationship between TCM constitution theory and modern thermophysiology. Accordingly, this multicenter, cross-sectional study leverages standardized IRT to explore body-surface temperature as a potential quantitative phenotype for TCM constitution. By mapping multisite thermal signatures across diverse regional thermal settings and acquisition sites in China, we aim to provide a descriptive framework linking traditional classification with infrared-derived temperature patterns for future hypothesis generation and quantitative phenotyping research. Our findings provide preliminary observational evidence for future studies on constitution-related differences in body-surface temperature patterns in older adults, offering a resource-efficient framework for large-scale population phenotyping.

## 2 Methods

### 2.1 Objectives

Primary objectives:

1. To characterize body-surface temperature phenotypes among older adults across broad regional thermal settings in China.
2. To assess associations between TCM constitution types and body-surface temperature phenotypes, with a specific focus on anatomical heterogeneity and site-specific spatial patterns.
3. To evaluate whether constitution-related site-specific thermal patterns were robust to center-level heterogeneity and not driven by individual raw acquisition sites.
  Secondary objectives:
4. To describe background thermal heterogeneity across broad regional settings and merged acquisition centers.
5. To quantify the incremental explanatory value of center adjustment, body-composition-related covariates, and TCM constitution for five major summary thermal metrics.
6. To describe the distribution of TCM constitution types across broad regional thermal settings.

### 2.2 Study design and setting

This population-based, multicenter observational study was conducted between September 2021 and June 2024 in community-based health examination settings across China. IRT data were collected from 13 raw acquisition sites: Hebei 1, Hebei 2, Hebei 3, Hebei 4, Hubei 1, Hubei 2, Hubei 3, Shenzhen 1, Shenzhen 2, Chengdu, Xinjiang, Zhejiang, and Changchun.

For the primary constitution-temperature analysis, raw acquisition sites were grouped into seven merged acquisition centers: Hebei, Hubei, Shenzhen, Chengdu, Xinjiang, Zhejiang, and Changchun. The merged-center structure was used as the main location-adjustment variable to account for center -level heterogeneity while avoiding over-parameterization from small raw sites. For descriptive background analysis, sites were further classified into three broad regional thermal settings—cold/northern, transitional/middle, and warm/southern—to characterize large-scale thermal heterogeneity. Raw acquisition site was retained for quality-control checks, alternative center-adjustment sensitivity analysis, and leave-one-site-out robustness analysis.

IRT was performed using a unified protocol for participant preparation, measurement conditions, device calibration, and image acquisition. Temperature data were extracted from prespecified body-surface regions of interest. The main regional analysis focused on 12 predefined trunk regions, and supplementary acupoint-level analysis were conducted at selected trunk and sacral acupoints to refine anatomical localization of constitution-related thermal signals (**Figure 1**).

**Figure 1:**
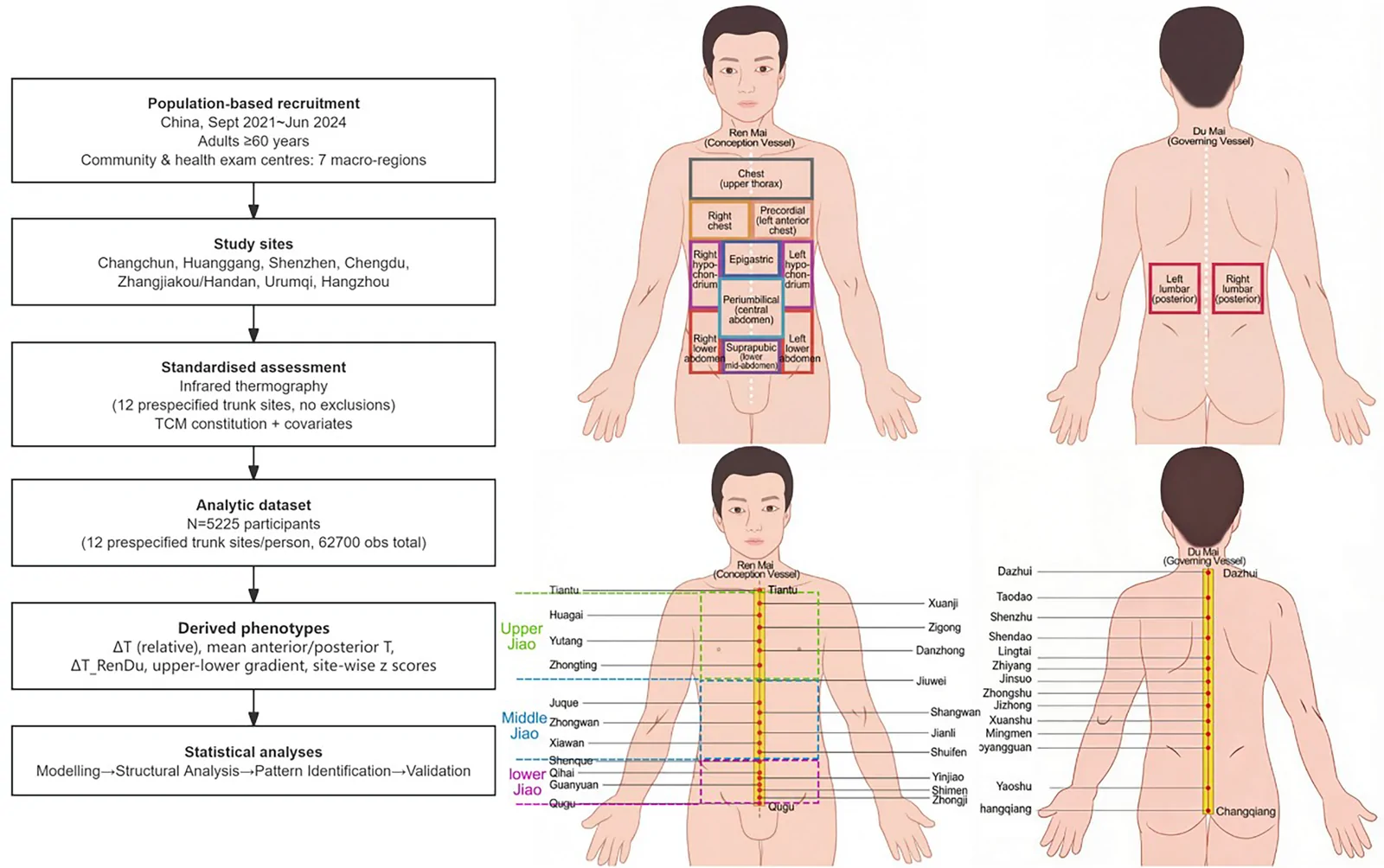
Study design, analytical framework, and infrared temperature measurement sites.

### 2.3 Participants

We consecutively recruited adults aged 60 years or older attending participating centers for routine health examinations or community assessments. Eligible participants were community-dwelling older adults capable of undergoing IRT and completing questionnaire-based assessments. The study was approved by the Ethics Committee of the Chinese Clinical Trial Registry, Beijing, China (Approval No. ChiECRCT20210494). Written informed consent was obtained from all participants.

### 2.4 Inclusion and exclusion criteria and sample selection

Eligible participants were community-dwelling older adults aged 60 years or above. All participants provided written informed consent and completed both the TCM constitution assessment and IRT examination.

Participants were included if they met the following criteria: (1) aged 60 years or older; and (2) willing to undergo IRT examination and able to provide written informed consent.

Participants were excluded if they met any of the following criteria: (1) mixed constitution types; (2) history of major trauma, major surgery, or physical disability; (3) diseases, medical conditions, or recent medication use that could substantially affect body temperature, autonomic function, peripheral circulation, or thermal imaging interpretation, including acute infection, fever, severe peripheral vascular disease, clinically diagnosed severe neuropathy, extensive skin lesions or scars in the imaging regions, or recent use of vasoactive medications that, in the investigators’ judgement, could interfere with thermal imaging; (4) receipt of acupuncture, moxibustion, cupping, massage, Gua Sha, or other physical therapies before the examination; (5) severe visual, hearing, speech, or psychiatric disorders that could interfere with completion of the questionnaire; (6) history of major illness, pregnancy, or childbirth within the past year, which might substantially alter constitution status; (7) incomplete questionnaire data or responses with obvious logical inconsistencies; or (8) IRT images that failed to meet the prespecified acquisition criteria.

### 2.5 Data collection and measurements

IRT was performed using a project-designated Intelligent Traditional Chinese Medicine Health Service System equipped with a portable DTA-301C infrared lens. Image acquisition was conducted in a controlled environment with ambient temperature maintained at 22–26 °C and relative humidity at 40%–60%, without direct airflow or direct heat/light exposure. Examinations were conducted between 08:00 and 11:00. Participants followed a standardized preparation protocol, including avoidance of alcohol, caffeine, strong tea, stimulating physical therapies, strenuous exercise, extreme thermal exposure, smoking, and cold or hot food/drinks within prespecified time windows. Before imaging, participants rested quietly for 15–20 min with the relevant skin regions exposed to allow acclimatization. Detailed procedures are provided in **Appendix 1** in **Supplementary Material**.

TCM constitution was assessed using the Constitution in Chinese Medicine Questionnaire for the Elderly. The questionnaire has demonstrated acceptable internal consistency, construct validity, and test–retest reliability in previous validation studies. According to the questionnaire scoring algorithm, individuals with a predominant Balanced constitution were further classified according to the predefined Balanced constitution criteria. In the present analysis, participants classified as “basic Balanced constitution” and “Balanced constitution” were combined into a single Balanced constitution category to ensure sufficient sample size and analytical consistency. Because some individuals in the combined Balanced category retained elevated scores for biased constitution tendencies, secondary constitution information was retained for descriptive overlap analysis. Participants with tied highest scores were classified according to the prespecified scoring algorithm of the questionnaire. This predominant constitution classification was used as the primary exposure variable in the analysis (Song et al., 2022). Detailed overlap patterns between predominant and secondary constitution categories are presented in **Supplementary Tables S8, S9**.

### 2.6 Outcomes and variables

Primary thermal outcomes were infrared-derived body-surface temperature phenotypes assessed at summary, regional, and acupoint levels. Summary metrics included anterior and posterior trunk mean temperatures, upper-lower body temperature difference, trunk-limb temperature difference, and anterior-posterior trunk temperature difference.

Regional trunk temperature was analyzed across 12 predefined trunk regions. For anterior trunk regions, ΔT was defined as the region-specific mean temperature minus the participant-level mean temperature across all anterior trunk regions. For posterior trunk regions, ΔT was defined analogously as the region-specific mean temperature minus the participant-level mean temperature across all posterior trunk regions. Because ΔT was calculated within each body-surface plane, values are mutually dependent within anterior and posterior regions and should be interpreted as relative spatial deviations rather than independent regional measures. This within-plane centring approach reduces between-person variation in overall surface temperature within each body-surface plane and emphasizes spatial temperature redistribution within the anterior and posterior regions.

Supplementary acupoint-level outcomes were defined as mean temperatures at selected trunk and sacral acupoints to refine the anatomical localization of regional thermal patterns.

The primary exposure was predominant TCM constitution type, with Balanced constitution as the reference category. Location-related variables included raw acquisition site, merged acquisition center, and broad regional thermal setting. Raw acquisition site was used for quality-control checks and sensitivity analysis; merged acquisition center was used as the primary location-adjustment variable; and broad regional thermal setting, classified as cold/northern, transitional/middle, or warm/southern, was used for descriptive background analysis.

Multivariable models adjusted for age, sex, body mass index, waist circumference, abdominal skinfold thickness, systolic blood pressure, and diastolic blood pressure.

Region-level analysis were excluded according to the predefined quality-control protocol and reacquired whenever possible. Images that still failed automated ROI extraction after reacquisition were excluded from the corresponding region-level analysis.

### 2.7 Statistical analysis

Baseline characteristics (**Table 1**; **Supplementary Tables**) were summarized using observed (non-imputed) data, with missingness reported. Continuous variables were expressed as mean (SD) or median (IQR), and categorical variables as n (%). Between- center variability in thermal metrics was assessed using intraclass correlation coefficients (ICCs) derived from one-way random-effects models. ICC analysis were performed using complete-case data because standard pooling methods for one-way ANOVA ICC estimates are not established.

**Table 1:** Characteristics of the study participants.

Characteristic	Overall (N = 5225)
Demographic characteristics	
Sex, n (%)	
Male	2050 (39·2)
Female	3175 (60·7)
Age group, years, n (%)	
60–69	3225 (61·7)
70–79	1698 (32·5)
≥80	302 (5·8)
Education level, n (%)	
Primary school or below	1695 (32·4)
Secondary school	2722 (52·1)
College or above	588 (11·3)
Anthropometric and physiological measures	
Body mass index, kg/m²	24·3 (3·3)
Waist circumference, cm	87·1 (9·5)
Systolic blood pressure, mmHg	135·7 (18·5)
Diastolic blood pressure, mmHg	80·4 (11·6)
Grip strength (dominant hand), kg †	21·3 (11·7)
Lifestyle factors	
Current smoking, n (%)	525 (10·0)
Current alcohol consumption, n (%)	641 (12·3)
Health status	
Any chronic disease, n (%) ‡	2886 (55·2)
Traditional Chinese medicine constitution	
Balanced constitution, n (%)	1803 (34·5)
Qi deficiency constitution, n (%)	176 (3·4)
Yang deficiency constitution, n (%)	793 (15·2)
Yin deficiency constitution, n (%)	713 (13·6)
Phlegm-dampness constitution, n (%)	1407 (26·9)
Damp-heat constitution, n (%)	85 (1·6)
Blood stasis constitution, n (%)	123 (2·4)
Qi stagnation constitution, n (%)	90 (1·7)
Special constitution, n (%)	35 (0·7)

Data are presented as mean (SD) for continuous variables and n (%) for categorical variables. Missing data: Education (n=220, 4·2%), Smoking (n=376, 7·1%), Alcohol (n=376, 7·1%), BMI (n=26, 0·4%), Blood pressure (n=278, 5·3%), Waist circumference(n=224, 4·2%), Grip strength(n=278, 5·3%).

†Grip strength measured in dominant hand.

‡Chronic diseases were defined as self-reported physician-diagnosed chronic conditions, including but not limited to hypertension, diabetes, coronary heart disease, and stroke.

Missing values in key covariates (body mass index, abdominal skinfold thickness, systolic blood pressure, and diastolic blood pressure) were handled using multiple imputation by chained equations (MICE) with predictive mean matching, generating five imputed datasets. Waist circumference was excluded to ensure a consistent body composition adjustment framework. All regression analyses were performed within each imputed dataset and combined using Rubin’s rules.

For analyses of the 12 predefined trunk regions, the data were arranged in long format, with anatomical region modelled as a categorical fixed effect. Constitution, anatomical region, and constitution-by-region interaction terms were included to estimate region-specific constitution effects relative to the Balanced constitution. Models were fitted by ordinary least squares within each imputed dataset, adjusting for merged acquisition center, broad regional setting, age, sex, body mass index, abdominal skinfold thickness, systolic blood pressure, and diastolic blood pressure. Within-participant correlation arising from repeated regional observations was accounted for using participant-clustered robust standard errors rather than random-effects modeling. Model assumptions were assessed by visual inspection of fitted-versus-residual plots and normal Q–Q plots. The outcome variable for regional analysis was ΔT, defined as the within-person deviation of each region from the participant-specific mean temperature within the corresponding anatomical plane, thereby characterizing relative within-plane spatial heterogeneity. Because ΔT values were centered within each anatomical plane, regional observations within participants were interpreted as relative spatial deviations rather than independent regional temperature measurements.

For the regional analysis, 96 constitution–region contrasts (12 regions × 8 unbalanced constitutions) were estimated, and pooled β estimates, standard errors, and 95% confidence intervals were obtained using Rubin’s rules. Multiple comparisons were controlled using the Benjamini–Hochberg false discovery rate procedure.

Relative importance of predictor blocks (regional setting, body composition, constitution, and demographics) was evaluated using Shapley (LMG) variance decomposition based on standardized linear models.

Model assumptions were assessed by visual inspection of fitted-versus-residual plots and normal Q–Q plots. No major systematic patterns or marked heteroscedasticity were observed. Mild-to-moderate departures from normality were present in the tails of some ΔT and differential-temperature models. Accordingly, robust or participant-clustered robust standard errors were used where appropriate. These diagnostics did not indicate violations likely to materially affect the primary population-average inferences.

Acupoint-level analyses were performed as exploratory analysis using absolute temperature as the outcome and the same covariate adjustment strategy, with heteroscedasticity-consistent (HC3) robust standard errors.

Three sensitivity analysis were conducted for the 12-region models. First, models were refitted using absolute regional mean temperature to assess consistency with ΔT-based results. Second, models were repeated using raw acquisition site instead of merged acquisition center. Third, leave-one-site-out analysis were performed by sequentially excluding each acquisition site to evaluate the influence of individual centers. Robustness was assessed based on directional consistency and stability of pooled effect estimates across analysis.

## 3 Results

### 3.1 Participant characteristics

**Figure 1** a total of 5,242 participants were initially recruited from the participating acquisition sites. After post-acquisition quality control, 17 participants were excluded due to incomplete or failed infrared temperature extraction, resulting in 5,225 participants included in the analytical dataset. For region-specific infrared thermal analysis, an additional 17 participants were excluded due to incomplete or failed extraction of region-of-interest temperature features, resulting in a valid dataset of 5,208 participants for these analyses. These exclusions were due to technical limitations in image feature extraction rather than missing covariate data. Among 5,225 participants, 2,823 participants (54.0%) were from cold/northern settings, 1,098 (21.0%) from transitional/middle settings, and 1,304 (25.0%) from warm/southern settings. Detailed overall characteristics are presented in **Table 1**; To improve clarity and readability of participant characteristics, we additionally provide a graphical summary of the sample structure (**Supplementary Figure S1**), including distributions of sex, age, lifestyle factors, and Traditional Chinese Medicine constitution across regions. **Supplementary Tables S1, S2** were reorganized to separately present continuous and categorical variables with consistent stratification by region and sex.

### 3.2 Background heterogeneity across broad regional settings

#### 3.2.1 Descriptive differences in thermal metrics across broad climatic groupings

After adjustment, Adjusted anterior and posterior surface temperatures decreased progressively from cold to warm settings (all p < 0.001; **Supplementary Table S3**, **Figure 2**). Participants from colder settings showed higher anterior and posterior surface temperatures, whereas those from warmer settings showed lower absolute temperatures. Upper-to-lower and trunk-to-limb differentials were also greatest in the cold group and attenuated in the middle and warm groups. By contrast, the anterior-to-posterior differential showed a smaller absolute effect size despite remaining statistically detectable. These findings indicate substantial background thermal heterogeneity across broad regional settings.

**Figure 2:**
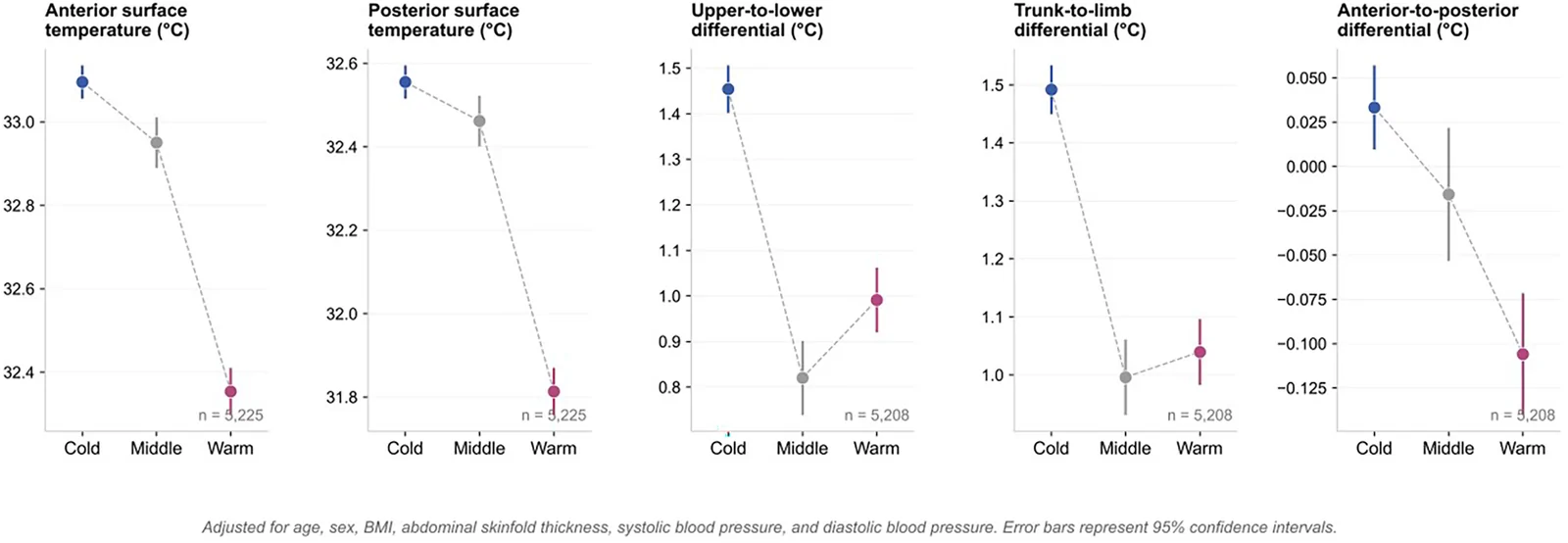
Adjusted means of thermal metrics across broad regional settings.

#### 3.2.2 Site-level variation in thermal patterns

Across the seven merged acquisition centers, center -level intraclass correlation coefficients ICCs ranged from 0.04 to 0.15, indicating modest center -level clustering (**Supplementary Table S4**, **Figure 3**). The highest center -level clustering was observed for Trunk-to-limb differential, whereas lower ICCs were observed for Anterior-to-posterior differential. These results indicate modest but non-negligible between- center thermal heterogeneity.

**Figure 3:**
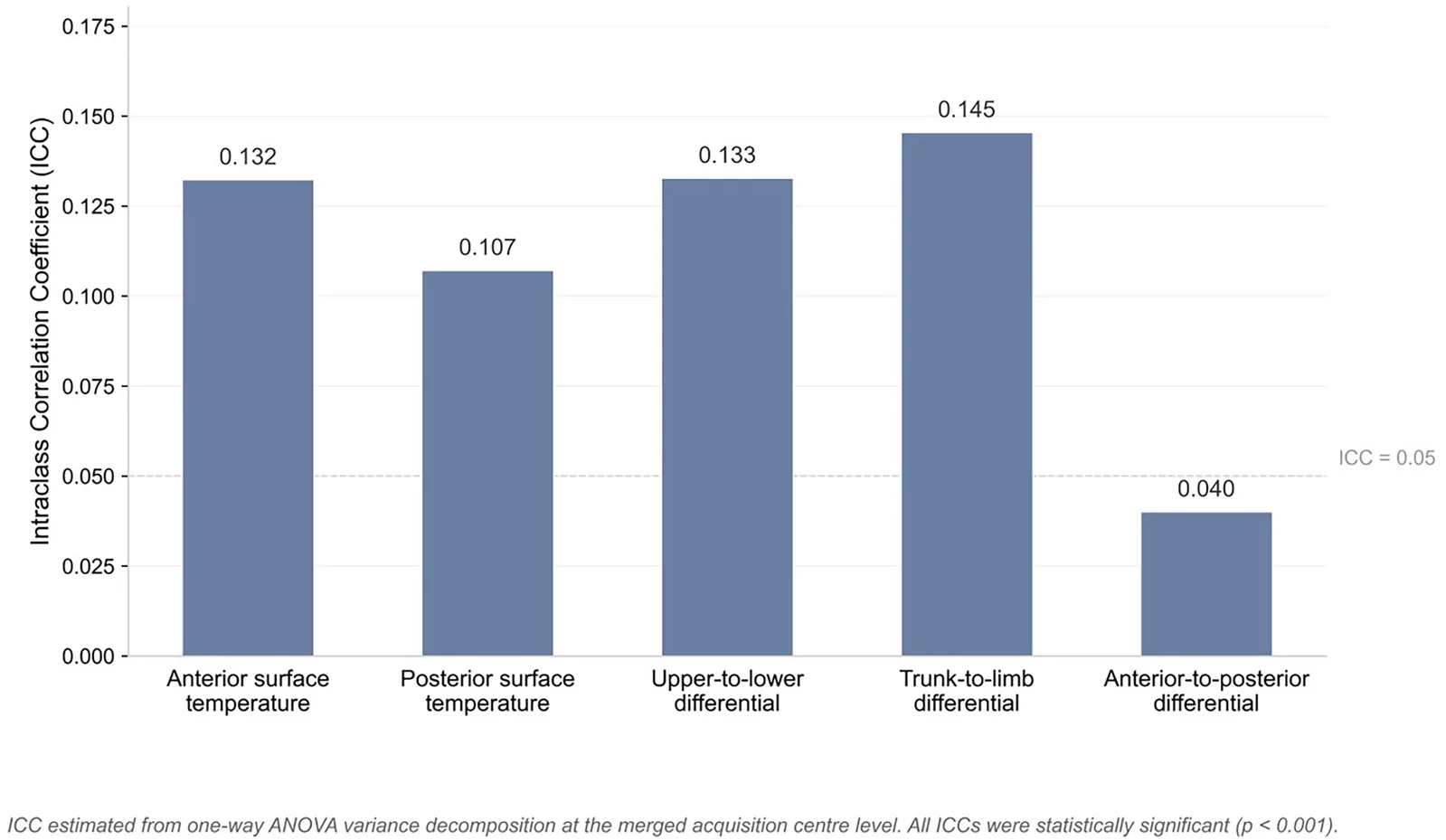
Intraclass correlation coefficients for thermal metrics across merged acquisition centers.

#### 3.2.3 Distribution of constitution types across settings

Constitution distribution differed significantly across broad regional settings (χ² = 168.32, df = 16, p < 0.001; **Table 2**). Phlegm-dampness constitution was more common in cold settings, whereas Yin-deficiency and Qi-deficiency showed relatively higher proportions in middle and warm settings, respectively.

**Table 2:** Distribution of constitution types across broad regional settings.

Constitution type	Cold, n (%)	Middle, n (%)	Warm, n (%)
Balanced	979 (34.7%)	381 (34.7%)	443 (34.0%)
Qi-deficiency	78 (2.8%)	18 (1.6%)	80 (6.1%)
Yang-deficiency	421 (14.9%)	144 (13.1%)	228 (17.5%)
Yin-deficiency	319 (11.3%)	216 (19.7%)	178 (13.7%)
Phlegm-dampness	875 (31.0%)	270 (24.6%)	262 (20.1%)
Damp-heat	39 (1.4%)	11 (1.0%)	35 (2.7%)
Qi stagnation	66 (2.3%)	24 (2.2%)	33 (2.5%)
Blood stasis	33 (1.2%)	29 (2.6%)	28 (2.1%)
Special diathesis	13 (0.5%)	5 (0.5%)	17 (1.3%)

Chi-square = 168.32, df = 16, p <0.001.

Percentages are column percentages within each broad regional setting. Chi-square test was performed on the full contingency table of constitution type by broad regional setting.

### 3.3 Relative contributions of regional setting, body composition, constitution, and demographic factors

Relative importance analysis was used to quantify overall variance attribution, whereas multivariable regression analysis were used to estimate region-specific associations.

Relative importance analysis demonstrated that regional setting was the dominant contributor to thermal variability across most outcomes (**Table 3**). The proportion of explained variance attributable to regional setting ranged from 42.7% in back surface temperature to 68.4% in upper-to-lower thermal differential, indicating a strong spatial structuring effect on body-surface thermal patterns.

**Table 3:** Relative importance of regional setting, body composition, constitution, and demographic factors in explaining thermal variability.

Outcome	N	R2	Region (%)	Body composition (%)	Constitution (%)	Age/sex (%)
front_avg	5225	0.196	57.56	34	3.632	4.808
back_avg	5225	0.222	42.988	42.969	5.584	8.459
upper_lower_diff	5208	0.147	69.164	17.941	1.976	10.92
trunk_limb_diff	5208	0.182	62.918	25.273	3.051	8.758
front_back_diff	5208	0.046	67.473	21.293	10.894	0.34

Body composition represented the second largest source of variability for most thermal metrics, accounting for 18.8%–43.2% of explained variance. Notably, body composition slightly exceeded regional contribution for back_avg (43.2% vs. 42.7%), suggesting a stronger individual physiological influence in posterior surface temperature.

Constitutional factors contributed relatively smaller proportions of variance overall (2.0%–10.9%), but showed relatively higher importance in front-back differential compared with other metrics, indicating a potential role in mediating anterior–posterior thermal balance.

Age and sex consistently explained the smallest proportion of variance across all outcomes (0.4%–10.8%), although their contribution was more pronounced in upper-to-lower and trunk-to-limb differentials.

These findings were robust across multiple imputed datasets (**Supplementary Tables S5–S7**), with consistent ranking of regional setting as the primary determinant of thermal variability.

### 3.4 Associations between TCM constitution and local thermal phenotypes

#### 3.4.1 Regional 12-zone ΔT patterns

Using a multiple imputation framework with Rubin’s pooling, constitution-associated differences in regional ΔT were evaluated across 12 predefined trunk regions (**Figure 4**, **Supplementary Table S10**). A heterogeneous pattern of constitution-specific thermal deviations was observed, with significant associations concentrated in selected anatomical sites. At the upper thorax, Qi-deficiency, Yang-deficiency, and Yin-deficiency showed small positive but non-significant associations.

**Figure 4:**
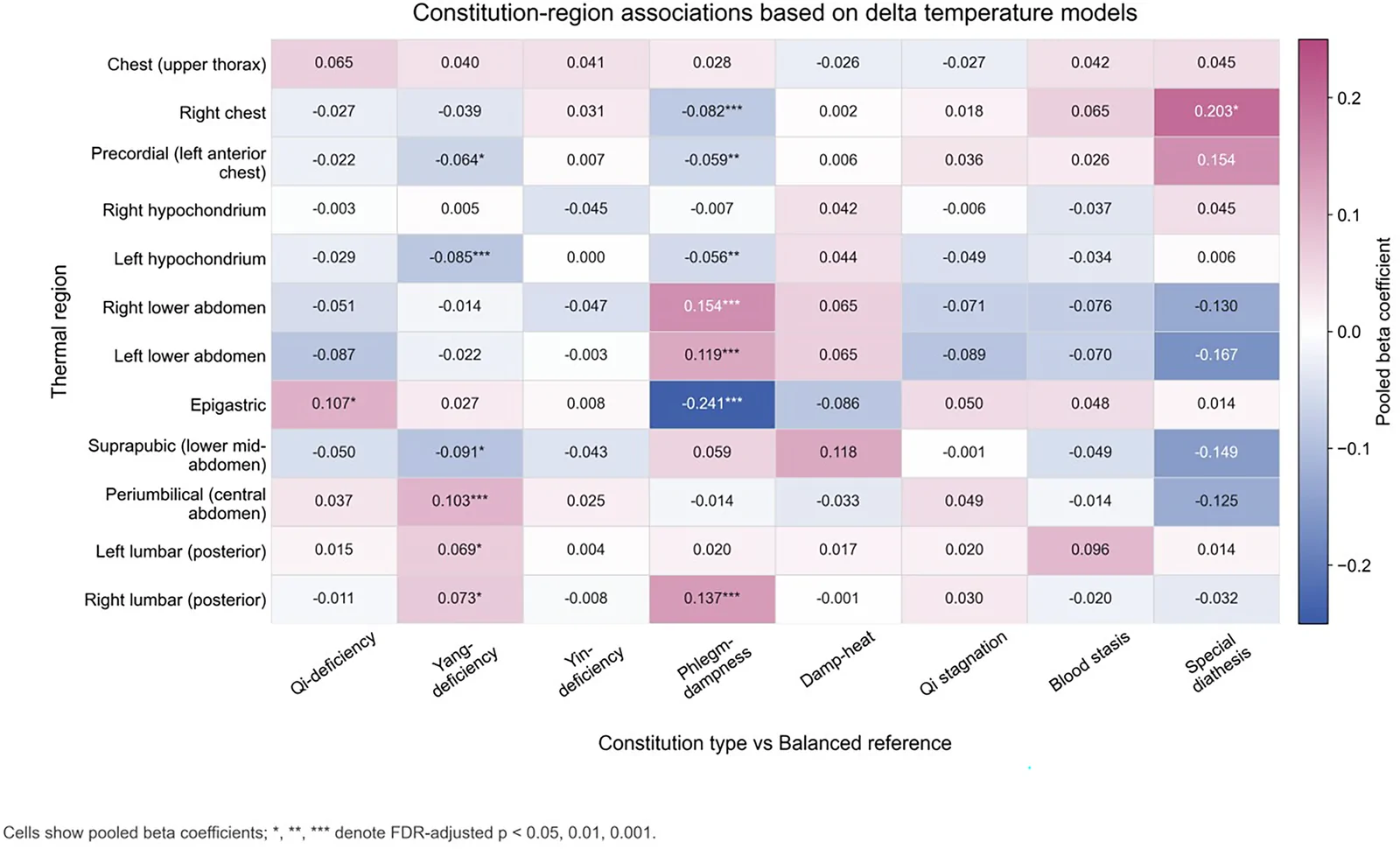
Site-specific differences in relative trunk temperature by TCM constitution. Cells show pooled adjusted β coefficients for each constitution type relative to the Balanced constitution across 12 trunk regions. ΔT was calculated separately within anterior and posterior planes as the deviation from the participant-specific mean temperature of the corresponding plane. Models were adjusted for broad geographic region, merged acquisition center, age, sex, BMI, abdominal skinfold thickness, systolic blood pressure, and diastolic blood pressure; estimates were pooled across multiple imputed datasets using Rubin’s rules. Asterisks denote FDR-adjusted significance (*p < 0.05, **p < 0.01, ***p < 0.001). ΔT represents within-plane relative temperature distribution rather than absolute regional temperature.

In contrast, more pronounced constitution-associated differences were observed in lateral thoracic regions. At the right chest, Phlegm-dampness was significantly associated with reduced ΔT (β = −0.082, FDR p < 0.001), while Special diathesis showed a significant positive association (β = 0.203, FDR p = 0.019). At the precordial (left anterior chest) region, significant negative associations were observed for Yang-deficiency (β = −0.064, FDR p = 0.013) and Phlegm-dampness (β = −0.058, FDR p = 0.003).

In the hypochondriac region, significant negative associations were consistently observed on the left side, where Yang-deficiency (β = −0.085, FDR p < 0.001) and Phlegm-dampness (β = −0.055, FDR p = 0.002) showed robust reductions in ΔT. In contrast, no FDR-significant associations were observed in the right hypochondrium.

In the abdominal region, constitution-associated thermal patterns were more spatially differentiated. At the epigastric region, Qi-deficiency (β = 0.107, FDR p = 0.037) showed a significant positive association, whereas Phlegm-dampness exhibited a strong negative association (β = −0.24, FDR p < 0.001). At the suprapubic region, Yang-deficiency was significantly negatively associated with ΔT (β = −0.091, FDR p = 0.019), while Phlegm-dampness showed a positive association (β = 0.059, FDR p = 0.082, nominal). At the periumbilical region, only Yang-deficiency demonstrated a robust positive association (β = 0.103, FDR p < 0.001), whereas other constitutions showed no significant effects after correction.

In the lower abdominal region, the most consistent and statistically robust associations were observed for Phlegm-dampness. Both the right lower abdomen (β = 0.154, FDR p < 0.001) and left lower abdomen (β = 0.120, FDR p < 0.001) showed significant positive deviations in ΔT. Qi-deficiency and Special diathesis showed weaker and non-significant associations after multiple-testing correction.

Posterior lumbar regions demonstrated more localized but consistent effects. At the left lumbar region, Yang-deficiency was significantly positively associated with ΔT (β = 0.069, FDR p = 0.019). At the right lumbar region, both Yang-deficiency (β = 0.073, FDR p = 0.019) and Phlegm-dampness (β = 0.137, FDR p < 0.001) showed significant positive associations.

Overall, the results indicate that constitution-associated thermal differences are not uniformly distributed across the trunk but instead exhibit region-specific patterns, with the most pronounced effects observed for Phlegm-dampness and Yang-deficiency constitutions. The concentration of significant findings in several constitutions may reflect their higher representation in the study population, which increases statistical power for detecting stable effects within these groups.

#### 3.4.2 Acupoint-level refinement of constitution-related thermal patterns

Acupoint analysis used absolute temperature rather than ΔT because the objective was to localize regional thermal signals to anatomically discrete sites, whereas ΔT was designed to characterize within-plane spatial redistribution at the regional level.

To further localize the regional thermal patterns identified in the 12-region analysis, acupoint-level analysis were performed using absolute acupoint temperatures as outcomes (**Supplementary Table S11**). Overall constitution effects were first evaluated at each acupoint using pooled multivariate Wald tests, with false discovery rate (FDR) correction applied across acupoints. Only acupoints demonstrating significant overall constitution effects were subsequently examined using *post hoc* constitution-specific contrasts.

After FDR correction, significant overall constitution effects were observed at two sacral acupoints: Shangliao (BL31) (FDR-adjusted P = 0.038) and Ciliao (BL32) (FDR-adjusted P = 0.024), whereas no other evaluated acupoints showed significant overall constitutional differences.

*Post hoc* comparisons within these two significant acupoints showed that participants with Phlegm-dampness constitution exhibited significantly lower absolute temperatures than those with Balanced constitution at both BL31 (β = −0.162, FDR-adjusted P = 0.006) and BL32 (β = −0.170, FDR-adjusted P = 0.005). No other constitution-specific contrasts remained significant after multiple-testing correction (**Supplementary Table S11**).

### 3.5 Alternative center-adjustment sensitivity analysis

Replacing merged acquisition center with raw acquisition site produced highly consistent effect estimates across constitution–region contrasts. All FDR-significant associations identified in the primary analysis remained significant with identical effect directions, although several additional nominal associations were observed, none remained significant after FDR correction. These findings indicate that the primary site-specific results were robust to the choice of location-adjustment variable (**Supplementary Table S12**).

### 3.6 Robustness analysis (leave-one-site-out)

In leave-one-site-out analysis across the 13 raw acquisition sites, the main findings were directionally robust. All Phlegm-dampness associations across the eight key regions retained the same direction in every leave-one-site-out iteration. For Yang-deficiency, all full-model significant associations remained directionally consistent, while only the weaker Left lower abdomen and Right lower abdomen associations showed a direction change in one iteration. The Epigastric Qi-deficiency association was also directionally stable. These results suggest that the observed constitution–region associations were not driven by any single raw acquisition site (**Figure 5**).

**Figure 5:**
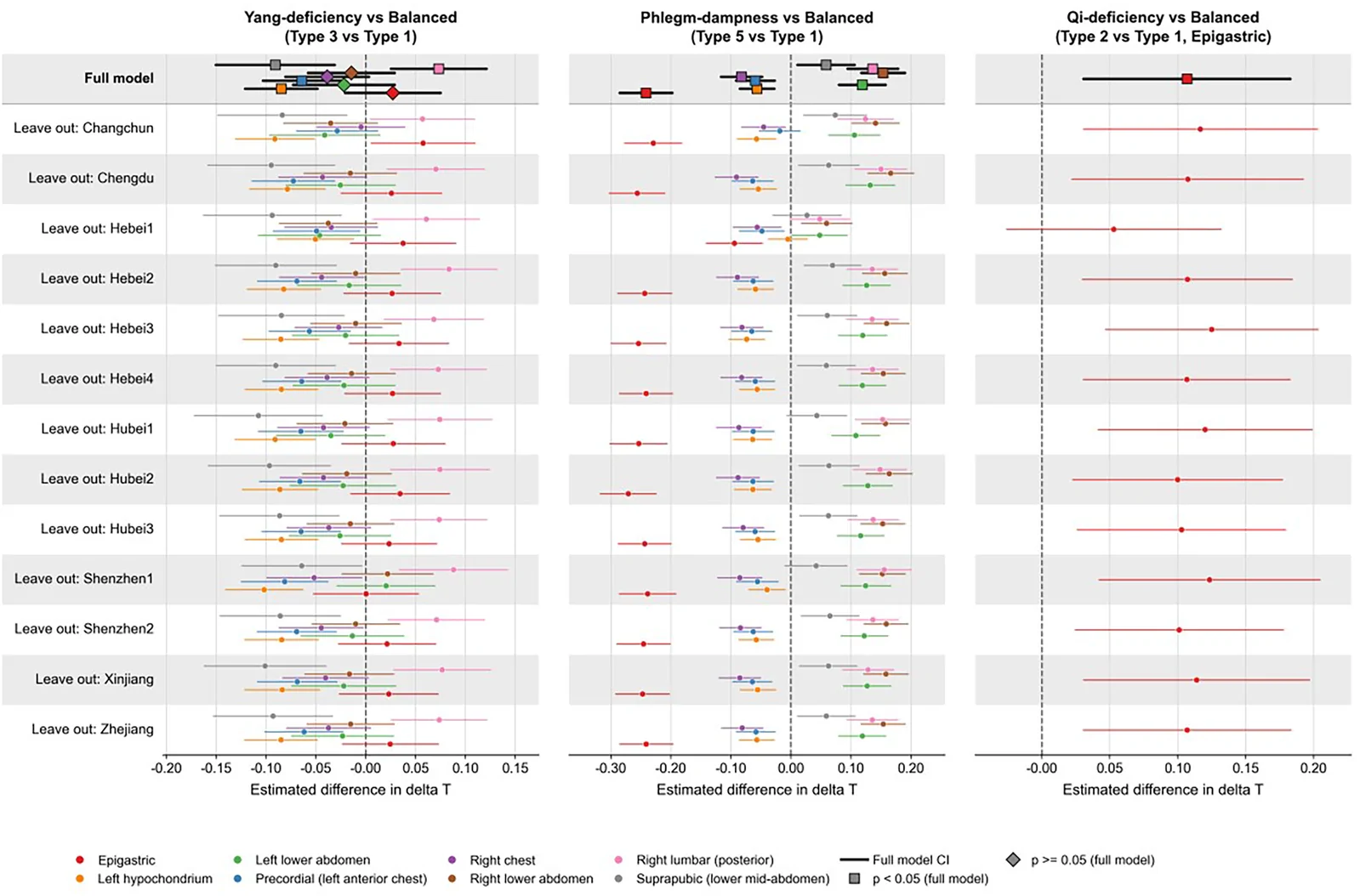
Leave-one-site-out robustness of key site-specific differences in relative skin temperature by constitution type.

## 4 Discussion

In this multicenter study of older adults across diverse regional settings in China, we characterized infrared-derived body-surface temperature patterns and examined their associations with Traditional Chinese Medicine (TCM) constitution types. Three main observations emerge. First, constitution-related differences were expressed primarily as spatial variation in surface temperature distribution rather than uniform shifts in overall temperature level. This was supported by the significant constitution-by-region interaction, indicating that constitution-related temperature differences varied across anatomical regions rather than acting as a global thermal shift. Second, constitution-related signals were localized to specific anatomical regions, predominantly involving abdominal, posterior lumbar, and selected thoracic regions, whereas most constitution–region combinations showed minimal differences. Third, although statistically detectable, the magnitude of observed differences was modest and directionally consistent across multiple sensitivity analysis, suggesting that these findings represent subtle but reproducible surface thermal phenotypes rather than clinically apparent thermal abnormalities.

A key methodological feature of this study was the use of relative temperature (ΔT) to characterize within-person spatial temperature distribution. By centring regional temperature values within anterior and posterior body-surface planes, ΔT reduces between-person variability in overall thermal status and highlights relative spatial redistribution. This approach is particularly relevant for multicenter population studies because absolute skin temperature is influenced by environmental exposure, ambient conditions, measurement procedures, and individual physiological differences. Previous infrared thermography studies have emphasized that skin temperature measurements are affected by multiple methodological factors, including camera characteristics, ROI definition, image analysis procedures, acclimatization time, and environmental conditions (Lahiri et al., 2012; Ludwig et al., 2014; Fernández-Cuevas et al., 2015). Therefore, within-person relative indices may provide complementary information to absolute temperature measurements by reducing the influence of between-subject variability.

The magnitude of the observed constitution-related differences should also be interpreted in the context of expected methodological and biological variability in infrared thermography. In the present study, significant regional differences ranged approximately from 0.08 to 0.27 °C. The smallest observed differences approached the magnitude of variability reported for some thermographic measurement procedures, whereas the larger differences exceeded typical repeatability estimates under standardized conditions and remained directionally consistent across absolute-temperature analysis, alternative center-adjustment strategies, and leave-one-site-out analysis. These findings suggest that the detected associations are unlikely to be explained solely by random measurement noise, although the effect sizes remain small. Importantly, absolute camera accuracy and measurement repeatability represent different concepts; although camera accuracy may be substantially larger than the observed effects, relative comparisons within the same thermal image generally demonstrate higher reproducibility under controlled acquisition conditions (Ammer, 2008; Ludwig et al., 2014). Accordingly, the present ΔT-based findings should be interpreted as subtle spatial organization of surface temperature rather than evidence of abnormal thermal states.

From a physiological perspective, infrared thermography reflects skin temperature, which represents an integrated consequence of cutaneous blood flow, tissue characteristics, autonomic regulation, and environmental heat exchange rather than a direct measurement of core thermoregulation. Skin temperature is inherently heterogeneous across anatomical sites because of differences in vascular anatomy, tissue composition, and local metabolic characteristics (Nilsson, 1987; Charkoudian, 2003; Charkoudian, 2010). The abdomen and posterior trunk are characterized by differences in soft tissue composition, subcutaneous fat distribution, and regional vascular organization, which may contribute to variation in local heat exchange and skin temperature regulation. These anatomical and physiological features may partly explain why constitution-associated signals, if present, are preferentially detected in these regions. Previous physiological studies have demonstrated that ageing is accompanied by reduced peripheral vascular responsiveness and altered thermoregulatory capacity, contributing to changes in skin perfusion and regional temperature regulation (Castellani and Young, 2016; Balmain et al., 2018). Age-related changes in thermoregulation are also region-dependent. Recent experimental studies have demonstrated that ageing alters sweating responses across different body regions during passive heat stress, suggesting that regional physiological differences contribute to individual variation in heat dissipation capacity (Kenney and Munce, 2003). These findings highlight that regional surface temperature patterns may provide complementary information for understanding age-related thermal adaptation and interactions with environmental conditions. Compared with the larger thermal changes observed during experimental heat or cold challenges, the differences observed in the present resting population represent subtle spatial redistribution rather than marked thermoregulatory dysfunction.

Beyond the context of constitution-related research, the regional surface temperature patterns characterized in this study may also provide exploratory information relevant to broader fields of geriatric thermal physiology and environmental health. Older adults exhibit age-related alterations in thermoregulation, including reduced sweating capacity, altered peripheral vascular responses, and changes in the relationship between skin temperature, thermal sensation, and core temperature regulation. Recent studies have highlighted the need for age-specific thermal comfort models because older adults may experience different physiological responses to environmental heat exposure compared with younger populations (Charkoudian, 2003; Li et al., 2026). In this context, large-scale characterization of regional body-surface temperature patterns may provide complementary phenotypic information for future studies investigating thermal comfort, environmental adaptation, and personalized thermal management in ageing populations.

The regional distribution of constitution-related thermal differences in the present study also showed similarities with previous constitution-oriented infrared thermography research conducted in China, although direct comparisons should be made cautiously because previous studies differed substantially in age groups, disease status, constitution classification procedures, imaging protocols, and analytical approaches. Several studies have repeatedly identified the abdomen, lower abdomen, lumbar region, and other central trunk areas as constitution-sensitive regions. For example, ΔT-based studies have reported constitution-associated thermal redistribution involving abdominal and lumbar areas among individuals with Yang-deficiency or Phlegm-dampness constitutions (Wang, 2017; Zhou et al., 2017; Wang, 2018; Zhang et al., 2018). These previous observations are broadly compatible with our findings that constitution-associated differences were concentrated in abdominal and posterior trunk regions. However, the direction and magnitude of thermal deviations have not been fully consistent across studies. This variation may reflect differences in population characteristics, physiological status, environmental conditions, and the complex nature of constitution classification. Therefore, constitution-related thermal patterns should be interpreted as regional phenotypic tendencies rather than fixed anatomical temperature signatures.

The use of relative temperature metrics in previous constitution-oriented studies also provides methodological context for the present findings. Several Chinese studies have applied ΔT-based approaches, using reference regions such as the trunk average temperature or other anatomical regions, to describe constitution-related thermal organization rather than absolute temperature differences (Hou et al., 2011; Wang, 2018). Similar to these studies, our results suggest that constitution-associated differences, if present, are expressed primarily as spatial redistribution rather than uniform elevation or reduction of body-surface temperature. However, unlike previous studies that were often performed in relatively small samples or selected disease populations, the present study provides evidence from a large multicenter cohort of community-dwelling older adults.

The substantial heterogeneity observed across regional and acquisition settings further underscores the context-dependent nature of surface temperature measurements. Even under standardized protocols, residual variability related to environmental exposure, behavioral factors, and local measurement conditions cannot be fully excluded. Previous studies have demonstrated that indoor thermal environments and acclimatization processes can substantially influence human skin temperature responses (Song et al., 2020). In our study, center-level clustering was modest (ICC 0.04–0.15), suggesting that acquisition setting contributed to thermal variability but accounted for only a limited proportion of overall variation after adjustment. Although center adjustment and sensitivity analysis reduced potential environmental influences, they cannot completely eliminate them. Therefore, infrared-derived thermal patterns should be interpreted within their environmental and procedural context rather than as purely intrinsic individual characteristics.

Although the present findings were robust across multiple sensitivity analysis, they should be interpreted in light of several limitations, which are discussed in detail below. First, the cross-sectional design precludes causal inference and does not allow assessment of temporal stability or predictive value. Second, constitution classification was based on a questionnaire-derived predominant type, which simplifies potentially overlapping or mixed constitution profiles. Although the Constitution in Chinese Medicine Questionnaire has demonstrated acceptable reliability and validity, questionnaire-based classification may still be influenced by reporting bias, cultural context, and temporal variation. Furthermore, constitution categories may not represent discrete biological entities, and the applicability of these classifications beyond the cultural context in which they were developed requires further investigation. Third, although the large sample size enabled detection of small differences, statistical significance should be interpreted alongside effect size, biological plausibility, and reproducibility.

Taken together, the present findings provide a quantitative framework for examining associations between infrared-derived thermal phenotypes and constitution classification. The present findings should be interpreted as observational evidence of constitution-related spatial temperature variation rather than mechanistic confirmation or clinical validation. Future longitudinal studies integrating physiological and environmental measurements will help clarify the biological basis and broader relevance of these thermal phenotypes.

## 5 Limitations and future directions

While this multicenter study provides novel evidence on constitution-related body-surface thermal phenotypes, several limitations should be considered. First, the study was restricted to older adults in China, which may limit generalizability to other age groups, ethnicities, and climatic contexts. Second, although TCM constitution was assessed using a previously validated questionnaire and constitution is generally regarded as a relatively stable individual characteristic, classification remained questionnaire-based and was based on self-reported and investigator-assisted assessment. Consequently, classification may still be influenced by reporting bias, cultural context, or temporal variation. In addition, constitution was analyzed as the predominant type according to the highest score rather than by explicitly modeling mixed or overlapping constitution profiles. Therefore, some degree of misclassification and simplification of constitution heterogeneity cannot be excluded. The applicability of questionnaire-derived constitution classification to populations outside the cultural context in which it was developed also requires further evaluation. Third, although IRT acquisition was standardized through controlled ambient temperature and humidity, avoidance of direct airflow, prespecified participant preparation, and a 15–20 min acclimatization period, IRT measures surface rather than core temperature. Residual variability related to unmeasured short-term environmental exposure, medication effects, recent behaviors, or individual microcirculatory status cannot be fully excluded. Although participants using medications judged to substantially affect vascular tone or thermal imaging interpretation were excluded, commonly used chronic disease medications in older adults may still subtly influence peripheral circulation, metabolic status, or autonomic regulation and contribute to residual variability in skin temperature. Fourth, despite center adjustment and leave-one-site-out analysis, uneven sample sizes across raw acquisition sites and broad regional settings may have affected the precision of some site-specific estimates. Fifth, no formal *a priori* power calculation was performed because this was an exploratory multicenter observational study, and the final sample size was determined by participant availability during the study period. Given the large sample size and repeated regional measurements, the study was designed primarily to estimate small population-level differences with high precision rather than to confirm a prespecified hypothesis regarding a clinically meaningful effect size. Although the large sample size enabled estimation of small constitution-related thermal differences, statistical significance should be interpreted alongside effect size, confidence intervals, spatial coherence, and robustness analysis. Finally, the cross-sectional design identifies statistical associations and generate testable hypotheses regarding underlying biological processes, but does not establish causality, clinical utility, or diagnostic performance. Because this study did not evaluate diagnostic classification, prediction performance, sensitivity, specificity, or outcome prediction, the observed thermal patterns should not be interpreted as validated biomarkers or screening tools.

Future studies should validate these findings in longitudinal and outcome-linked cohorts, explicitly assess mixed or multi-label constitution profiles, and incorporate more comprehensive environmental and behavioral exposure monitoring. Expanding anatomical coverage to the head, limbs, and additional acupoints may help develop more complete thermal maps. Integration with multi-omics, metabolic, vascular, or microcirculatory measures may further clarify the biological mechanisms, temporal stability, and potential predictive value of constitution-related thermal phenotypes.

## 6 Conclusion

This multicenter study provides a multicenter analytical framework for population-level investigation of spatial body-surface temperature phenotypes in older adults. Background regional setting, center adjustment, and body-composition-related covariates accounted for most explainable variability in summary thermal metrics, whereas TCM constitution was associated with modest, reproducible, and spatially organized thermal differences expressed mainly as site-specific spatial deviations rather than uniform shifts in overall temperature. These findings support the use of spatial thermal phenotyping as an observational framework for studying constitution-related variation in older adults under standardized infrared thermography protocols.

## Data Availability

The original contributions presented in the study are included in the article/**Supplementary Material**, further inquiries can be directed to the corresponding author/s.
