# Supplementary material for: Population-level spatial patterns of body-surface temperature in older adults: a multicenter infrared thermography study across diverse environmental settings
Source: Front Physiol. 2026 Oct 28;17:1870629. doi: 10.3389/fphys.2026.1870629 (PMC13751364; doi:10.3389/fphys.2026.1870629)
Supplement: Supplementary file 1 [file Table1.docx]

Supplementary Material 1: Data Collection

1. Participants and Ethics

The study was conducted following ethical approval from the Chinese Registered Clinical Trial Ethics Committee (ChiCTR2100053726). Participants were recruited on a voluntary basis after providing informed consent. The research process strictly adhered to medical ethical principles to ensure participant privacy and data security.

2. Data Collection: Content and Methods

This study employed a cross-sectional design, integrating questionnaires, physical examinations, and infrared thermography (IRT) for data collection. Key data points included sociodemographics, Traditional Chinese Medicine (TCM) constitution, lifestyle factors, and infrared thermal data.

2.1 Sociodemographics and Lifestyle Data Information was gathered via a unified data collection app or the "Questionnaire Star" platform, with on-site guidance from investigators. Collected items included:Sociodemographics: Sex, date of birth, education level, previous occupation, marital status, living situation, social engagement, and allergy history.

Lifestyle Factors: Smoking status, alcohol consumption, and dietary preferences. These data were utilised to characterise participant profiles and served as covariates in subsequent statistical analyses.

2.2 Physical Examination and Vital Signs Standardised measurements were performed by trained investigators. Indices included height, weight, waist circumference, abdominal skinfold thickness, systolic and diastolic blood pressure, and respiratory rate. All measurements were completed within a unified timeframe to minimise measurement error.

2.3 TCM Constitution Assessment TCM constitutions were identified using the TCM Constitution Questionnaire for the Elderly. Participants completed the questionnaire independently under investigator guidance. Types were classified into Balanced constitution and eight biased types (Qi-deficiency, Yang-deficiency, Yin-deficiency, Phlegm–dampness, Dampness–heat, Blood-stasis, Qi-stagnation, and Special-diathesis). This served as the primary exposure variable.

2.4 Infrared Thermography (IRT)

A. Equipment and acquisition system

Infrared thermography was performed using the Intelligent Traditional Chinese Medicine Health Service System provided by Beijing Eagle Eye Intelligent Health Technology Co., Ltd. Thermal images were acquired using a portable DTA-301C infrared lens. All participating sites used the same project-designated equipment and standardised acquisition protocol. After image acquisition, infrared data were centrally exported through the national networked infrared imaging data platform and matched with participants’ TCM constitution data for subsequent analysis.

B. Imaging room and camera positioning requirements

The imaging room was required to provide sufficient longitudinal space to meet the maximum acquisition distance. The acquisition distance included both half-body and whole-body imaging distances. For half-body imaging, including anterior and posterior chest views, the required distance was 210–220 cm. For whole-body imaging, including anterior and posterior whole-body views, the required distance was 260–270 cm. Considering the additional space occupied by the column-mounted device body, the longitudinal length of the imaging room was required to be at least 350 cm. The default installation height of the infrared lens was 115 ± 3 cm. During image acquisition, the lens height was adjusted according to the participant’s height to ensure that imaging requirements were met.

C. Environmental control

Image acquisition was performed in a dedicated examination room under controlled environmental conditions. Ambient temperature was maintained at 22–26°C and relative humidity at 40%–60%. The examination room was kept free from direct airflow; when air conditioning was used, participants were not exposed to direct air currents. Cold light sources were used to minimise interference from direct illumination or external heat sources.

D. Timing of image acquisition

Examinations were conducted between 08:00 and 11:00.

Figure 1. Schematic illustration of the infrared thermography acquisition environment

Figure 2 Schematic of infrared lens installation height (H)

Participants were instructed to follow a standardised preparation protocol before infrared image acquisition. On the day before examination, participants were asked to avoid alcohol, coffee, strong tea, and other stimulating beverages, and to avoid electrotherapy, moxibustion, heat therapy, cupping, or other stimulating physical therapies. Female participants were advised not to undergo imaging during menstruation. Participants were instructed to avoid strenuous exercise within 4 h before imaging and to avoid exposure to extremely cold or hot environments within 1 h before imaging. Within 1 h before imaging, participants were also asked to avoid consuming excessively cold or hot foods or drinks. Lactating women were asked to avoid breastfeeding within 1 h before imaging. Within 30 min before imaging, participants were instructed to avoid smoking, drinking water, and washing their hands with cold water. Participants were asked to report recent medication use, particularly chemotherapy drugs, hormonal medications, vasoconstrictors, and vasodilators.

Before image acquisition, participants were asked to report relevant medical history, recent examinations, and medication use, particularly chemotherapy drugs, hormonal medications, and vasoactive medications such as vasoconstrictors or vasodilators. After entering the examination room, participants rested quietly for 15–20 min to allow acclimatisation and stabilisation of body-surface temperature. If sweating was present, the skin was allowed to dry naturally before imaging. During the resting period, participants removed hats or clothing according to the body region to be examined and avoided tight clothing. They were instructed not to press, scratch, or rub the regions to be imaged. The skin surface was kept clean; topical patches, plasters, or ointments were removed whenever possible. Female participants were advised to avoid heavy make-up. Glasses, jewellery, and other accessories were removed before imaging, and participants were asked to empty the bladder before the examination.

Figure 3: Standardised acquisition postures for infrared thermography.

2.5 Thermal Partitioning and Metric Extraction In accordance with technical standards for TCM infrared thermography, the trunk was partitioned into specific regions of interest (ROIs):

1. Overall trunk regions (anterior and posterior).

2. Visceral projection areas: These regions comprise the Chest (upper thorax), Right chest, Precordial (left anterior chest), Epigastric, Periumbilical (central abdomen), Suprapubic (lower mid-abdomen), Right and Left hypochondrium, Right and Left lower abdomen, and Right and Left lumbar (posterior) regions.

3. Triple Jiao regions (upper, middle, and lower Jiao).

4. The Ren and Du meridian regions. Quantitative metrics, primarily mean temperature, were extracted for each ROI to compare thermal distribution patterns across different TCM constitutions

Figure 4: Schematic illustration of anterior and posterior trunk partitioning.

Figure 5: Schematic illustration of the Triple Jiao (Upper, Middle, and Lower) regions.

Figure 6: Schematic illustration of the Ren and Du meridians.

2.5.1 Image processing and quality control

Thermal image processing and region-of-interest (ROI) extraction were performed automatically using the built-in software of the Intelligent Traditional Chinese Medicine Health Service System. The software applies a proprietary anatomical recognition algorithm implemented by the manufacturer to automatically identify predefined anatomical regions and extract the corresponding mean temperature values. No manual ROI delineation or operator-dependent adjustment was performed; therefore, inter-rater reliability assessment was not applicable.

The infrared detector acquires raw thermal grayscale data, whereas pseudocolour thermal images are generated automatically by the system for visualisation only and are not used for temperature calculation or ROI identification.

All participating centres used the same project-designated imaging system with identical acquisition settings provided by the manufacturer. Automatic focusing was used throughout image acquisition, and no additional warm-up procedure was required before routine imaging according to the manufacturer's operating specifications. Following completion of participant recruitment at each acquisition centre, the infrared imaging lenses were returned to the manufacturer for routine calibration in accordance with the manufacturer's maintenance procedures.

Images with incomplete body-region coverage, obvious clothing or accessory occlusion, substantial motion artefacts, or unsuccessful automated ROI recognition were excluded according to the predefined quality-control protocol and reacquired whenever possible.

3. Quality Control

Strict measures were implemented to ensure data reliability:

1. All constitution assessments were performed by trained investigators from the National Institute of TCM Constitution and Preventive Medicine following a standardized assessment manual. Investigators completed centralized training before participant recruitment, and interviews were conducted in a structured face-to-face format to minimize inter-rater variability.

2. Designated equipment and standardised operational protocols were used across all sites.

3. Regular data audits were performed; thermal images failing to meet quality standards were promptly re-acquired.

4. Data auditing and feedback mechanisms were established to maintain data integrity and consistency.
