# Supplementary material for: Population-level spatial patterns of body-surface temperature in older adults: a multicenter infrared thermography study across diverse environmental settings
Source: Front Physiol. 2026 Oct 28;17:1870629. doi: 10.3389/fphys.2026.1870629 (PMC13751364; doi:10.3389/fphys.2026.1870629)
Supplement: Supplementary file 2 [file Table2.docx]

**Supplementary appendix 2. Additional analyses of participant characteristics, thermal phenotypes, and constitution classification**

**Participant characteristics**

| **Table S1. Continuous characteristics of participants stratified by region and sex** | | | | | | |
| --- | --- | --- | --- | --- | --- | --- |
| **Variable** | **Cold Male (n=1124)** | **Cold Female (n=1699)** | **Middle Male (n=440)** | **Middle Female (n=658)** | **Warm Male (n=486)** | **Warm Female (n=818)** |
| Body mass index (kg/m^2^) | 24.9 (3.1) | 24.8 (3.4) | 24.1 (3.2) | 23.5 (3.2) | 24.1 (3.1) | 23.6 (3.1) |
| Waist circumference (cm) | 90.2 (8.6) | 84.6 (8.5) | 90.9 (11.2) | 87.4 (10.3) | 88.6 (10.0) | 84.9 (9.6) |
| Systolic BP (mmHg) | 135.2 (16.3) | 132.6 (16.7) | 136.6 (19.5) | 136.8 (19.8) | 135.3 (20.1) | 132.5 (21.3) |
| Diastolic BP (mmHg) | 81.7 (10.0) | 78.3 (10.2) | 82.1 (11.2) | 80.5 (12.0) | 82.3 (13.1) | 80.7 (14.4) |
| Grip strength (kg) | 21.9 (8.0) | 14.1 (7.9) | 28.4 (12.2) | 21.4 (9.9) | 28.0 (15.9) | 18.4 (10.5) |

| **Table S2. Categorical characteristics of participants stratified by region and sex** | | | | | | |
| --- | --- | --- | --- | --- | --- | --- |
| **Sex, n (%)** |  |  |  |  |  |  |
| **Variable** | **Cold Male** | **Cold Female** | **Middle Male** | **Middle Female** | **Warm Male** | **Warm Female** |
| Male | 1124 (100) | — | 440 (100) | — | 486 (100) | — |
| Female | — | 1699 (100) | — | 658 (100) | — | 818 (100) |
|  |  |  |  |  |  |  |
| **Smoking status, n (%)** |  |  |  |  |  |  |
| **Category** | **Cold Male** | **Cold Female** | **Middle Male** | **Middle Female** | **Warm Male** | **Warm Female** |
| Current | 321 (28.6) | 18 (1.1) | 112 (25.5) | 16 (2.4) | 72 (14.8) | 4 (0.5) |
| Former | 158 (14.1) | 4 (0.2) | 58 (13.2) | 11 (1.7) | 70 (14.4) | 5 (0.6) |
| Never | 561 (49.9) | 1581 (93.1) | 270 (61.4) | 631 (95.9) | 272 (56.0) | 703 (85.9) |
|  |  |  |  |  |  |  |
| **Alcohol consumption, n (%)** |  |  |  |  |  |  |
| **Category** | **Cold Male** | **Cold Female** | **Middle Male** | **Middle Female** | **Warm Male** | **Warm Female** |
| Current | 479 (42.6) | 36 (2.1) | 143 (32.5) | 38 (5.8) | 123 (25.3) | 41 (5.0) |
| Former | 95 (8.5) | 6 (0.4) | 21 (4.8) | 4 (0.6) | 30 (6.2) | 6 (0.7) |
| Never | 484 (43.1) | 1561 (91.9) | 276 (62.7) | 616 (93.6) | 261 (53.7) | 665 (81.3) |
|  |  |  |  |  |  |  |
| **Chronic disease, n (%)** |  |  |  |  |  |  |
| **Variable** | **Cold Male** | **Cold Female** | **Middle Male** | **Middle Female** | **Warm Male** | **Warm Female** |
| Any chronic disease | 646 (57.5) | 999 (58.8) | 306 (69.5) | 443 (67.3) | 202 (41.6) | 290 (35.5) |
|  |  |  |  |  |  |  |
| **TCM constitution, n (%)** |  |  |  |  |  |  |
| **Constitution** | **Cold Male** | **Cold Female** | **Middle Male** | **Middle Female** | **Warm Male** | **Warm Female** |
| Balanced | 372 (33.1) | 607 (35.7) | 158 (35.9) | 223 (33.9) | 171 (35.2) | 272 (33.3) |
| Qi-deficiency | 22 (2.0) | 56 (3.3) | 4 (0.9) | 14 (2.1) | 32 (6.6) | 48 (5.9) |
| Yang-deficiency | 104 (9.3) | 317 (18.7) | 51 (11.6) | 93 (14.1) | 53 (10.9) | 175 (21.4) |
| Yin-deficiency | 106 (9.4) | 213 (12.5) | 71 (16.1) | 145 (22.0) | 65 (13.4) | 113 (13.8) |
| Phlegm-dampness | 460 (40.9) | 415 (24.4) | 130 (29.5) | 140 (21.3) | 122 (25.1) | 140 (17.1) |
| Damp-heat | 21 (1.9) | 18 (1.1) | 6 (1.4) | 5 (0.8) | 14 (2.9) | 21 (2.6) |
| Blood stasis | 18 (1.6) | 15 (0.9) | 7 (1.6) | 22 (3.3) | 8 (1.6) | 20 (2.4) |
| Qi stagnation | 16 (1.4) | 50 (2.9) | 9 (2.0) | 15 (2.3) | 14 (2.9) | 19 (2.3) |
| Special diathesis | 5 (0.4) | 8 (0.5) | 4 (0.9) | 1 (0.2) | 7 (1.4) | 10 (1.2) |
| Variables are grouped into demographic, lifestyle, health status, and Traditional Chinese Medicine constitution domains for clarity of presentation. Missing data were excluded from percentage calculations. | | | | | | |

**Regional thermal variation and centre effects**

| **Table S3. Adjusted means of thermal metrics across broad regional settings** | | | | | | |
| --- | --- | --- | --- | --- | --- | --- |
| **Variable** | **Cold, adjusted mean (95% CI)** | **Middle, adjusted mean (95% CI)** | **Warm, adjusted mean (95% CI)** | **F** | **P value** | **Analytic N** |
| Anterior surface temperature | 33.10 (33.06, 33.13) | 32.95 (32.89, 33.01) | 32.35 (32.30, 32.41) | 246.04 | <0.001 | 5225 |
| Posterior surface temperature | 32.56 (32.52, 32.59) | 32.46 (32.40, 32.52) | 31.81 (31.76, 31.87) | 252.34 | <0.001 | 5225 |
| Upper-to-lower differential | 1.45 (1.40, 1.50) | 0.82 (0.74, 0.90) | 0.99 (0.92, 1.06) | 91.63 | <0.001 | 5208 |
| Trunk-to-limb differential | 1.49 (1.45, 1.53) | 1.00 (0.93, 1.06) | 1.04 (0.98, 1.09) | 102.84 | <0.001 | 5208 |
| Anterior-to-posterior differential | 0.03 (0.01, 0.06) | -0.02 (-0.05, 0.02) | -0.11 (-0.14, -0.07) | 21.83 | <0.001 | 5208 |
| Adjusted means were estimated from linear regression models fitted separately in each MICE-PMM imputed dataset and pooled using Rubin's rules. Models included broad regional setting and were adjusted for age, sex, BMI, abdominal skinfold thickness, systolic blood pressure, and diastolic blood pressure, with covariates held at their analytic-sample means. Values are adjusted mean (95% confidence interval) in degrees C. F statistics and P values reflect pooled overall tests for broad regional setting. Analytic N: anterior and posterior surface temperature, n = 5,225; upper-to-lower differential, trunk-to-limb differential, and anterior-to-posterior differential, n = 5,208. | | | | | | |

| **Table S4. Centre-level intraclass correlation coefficients for thermal metrics** | | | | |
| --- | --- | --- | --- | --- |
| **Variable** | **ICC** | **F** | **P value** | **N** |
| Anterior surface temperature | 0.132 | 103.04 | <0.001 | 5,225 |
| Posterior surface temperature | 0.107 | 81.28 | <0.001 | 5,225 |
| Upper-to-lower differential | 0.133 | 103.11 | <0.001 | 5,208 |
| Trunk-to-limb differential | 0.145 | 114.51 | <0.001 | 5,208 |
| Anterior-to-posterior differential | 0.040 | 28.83 | <0.001 | 5,208 |
| ICC = intraclass correlation coefficient. ICC indicates the proportion of total variance attributable to between-centre clustering, estimated via one-way ANOVA variance decomposition at the merged acquisition centre level (k = 7 centres). F statistics and P values reflect the overall between-centre heterogeneity test. Complete-case analysis was applied for variables with missing values. | | | | |

**Multivariable models and variance decomposition**

| **Table S5. Fully adjusted multivariable regression coefficients for the five thermal metrics.** | | | | | | | |
| --- | --- | --- | --- | --- | --- | --- | --- |
| **Thermal metric** | **Predictor** | **β** | **SE** | **p value** | **95% CI lower** | **95% CI upper** | **N** |
| Anterior surface temperature | Intercept | 34.6508 | 0.2062 | <0.001 | 34.2466 | 35.0551 | 5225 |
| Anterior surface temperature | broad_region: middle vs cold | -0.4491 | 0.0719 | <0.001 | -0.5900 | -0.3083 | 5225 |
| Anterior surface temperature | broad_region: warm vs cold | -0.5880 | 0.0311 | <0.001 | -0.6490 | -0.5270 | 5225 |
| Anterior surface temperature | sex: female vs male | 0.0328 | 0.0273 | 0.230 | -0.0207 | 0.0863 | 5225 |
| Anterior surface temperature | Constitution: Qi-deficiency vs Balanced | -0.0365 | 0.0747 | 0.625 | -0.1828 | 0.1099 | 5225 |
| Anterior surface temperature | Constitution: Yang-deficiency vs Balanced | 0.0447 | 0.0401 | 0.265 | -0.0339 | 0.1234 | 5225 |
| Anterior surface temperature | Constitution: Yin-deficiency vs Balanced | 0.0371 | 0.0415 | 0.371 | -0.0441 | 0.1183 | 5225 |
| Anterior surface temperature | Constitution: Phlegm-dampness vs Balanced | -0.0251 | 0.0358 | 0.484 | -0.0952 | 0.0451 | 5225 |
| Anterior surface temperature | Constitution: Damp-heat vs Balanced | -0.1098 | 0.1035 | 0.289 | -0.3127 | 0.0930 | 5225 |
| Anterior surface temperature | Constitution: Qi stagnation vs Balanced | 0.1312 | 0.0869 | 0.131 | -0.0390 | 0.3015 | 5225 |
| Anterior surface temperature | Constitution: Blood stasis vs Balanced | 0.0392 | 0.1007 | 0.697 | -0.1582 | 0.2366 | 5225 |
| Anterior surface temperature | Constitution: Special diathesis vs Balanced | 0.0225 | 0.1591 | 0.887 | -0.2892 | 0.3343 | 5225 |
| Anterior surface temperature | Age | 0.0156 | 0.0023 | <0.001 | 0.0111 | 0.0201 | 5225 |
| Anterior surface temperature | BMI | -0.0759 | 0.0047 | <0.001 | -0.0851 | -0.0668 | 5225 |
| Anterior surface temperature | Abdominal skinfold thickness | -0.0048 | 0.0012 | <0.001 | -0.0071 | -0.0025 | 5225 |
| Anterior surface temperature | Systolic blood pressure | -0.0018 | 0.0009 | 0.059 | -0.0037 | 0.0001 | 5225 |
| Anterior surface temperature | Diastolic blood pressure | -0.0026 | 0.0014 | 0.069 | -0.0055 | 0.0002 | 5225 |
| Posterior surface temperature | Intercept | 34.3322 | 0.2078 | <0.001 | 33.9249 | 34.7396 | 5225 |
| Posterior surface temperature | broad_region: middle vs cold | -0.4995 | 0.0724 | <0.001 | -0.6414 | -0.3577 | 5225 |
| Posterior surface temperature | broad_region: warm vs cold | -0.6212 | 0.0313 | <0.001 | -0.6826 | -0.5598 | 5225 |
| Posterior surface temperature | sex: female vs male | -0.1877 | 0.0275 | <0.001 | -0.2417 | -0.1338 | 5225 |
| Posterior surface temperature | Constitution: Qi-deficiency vs Balanced | -0.0084 | 0.0752 | 0.911 | -0.1558 | 0.1390 | 5225 |
| Posterior surface temperature | Constitution: Yang-deficiency vs Balanced | 0.0930 | 0.0404 | 0.022 | 0.0137 | 0.1722 | 5225 |
| Posterior surface temperature | Constitution: Yin-deficiency vs Balanced | 0.1116 | 0.0417 | 0.008 | 0.0298 | 0.1934 | 5225 |
| Posterior surface temperature | Constitution: Phlegm-dampness vs Balanced | -0.0497 | 0.0360 | 0.168 | -0.1203 | 0.0209 | 5225 |
| Posterior surface temperature | Constitution: Damp-heat vs Balanced | -0.0510 | 0.1042 | 0.625 | -0.2552 | 0.1533 | 5225 |
| Posterior surface temperature | Constitution: Qi stagnation vs Balanced | 0.1890 | 0.0875 | 0.031 | 0.0175 | 0.3605 | 5225 |
| Posterior surface temperature | Constitution: Blood stasis vs Balanced | 0.1311 | 0.1014 | 0.196 | -0.0678 | 0.3299 | 5225 |
| Posterior surface temperature | Constitution: Special diathesis vs Balanced | 0.0356 | 0.1602 | 0.824 | -0.2784 | 0.3496 | 5225 |
| Posterior surface temperature | Age | 0.0188 | 0.0023 | <0.001 | 0.0143 | 0.0232 | 5225 |
| Posterior surface temperature | BMI | -0.0911 | 0.0047 | <0.001 | -0.1003 | -0.0819 | 5225 |
| Posterior surface temperature | Abdominal skinfold thickness | -0.0070 | 0.0012 | <0.001 | -0.0093 | -0.0046 | 5225 |
| Posterior surface temperature | Systolic blood pressure | -0.0006 | 0.0009 | 0.518 | -0.0025 | 0.0013 | 5225 |
| Posterior surface temperature | Diastolic blood pressure | -0.0030 | 0.0015 | 0.050 | -0.0061 | 0.0000 | 5225 |
| Upper-to-lower differential | Intercept | 1.5787 | 0.2550 | <0.001 | 1.0790 | 2.0785 | 5208 |
| Upper-to-lower differential | broad_region: middle vs cold | -0.1326 | 0.0898 | 0.140 | -0.3087 | 0.0434 | 5208 |
| Upper-to-lower differential | broad_region: warm vs cold | -0.0193 | 0.0389 | 0.620 | -0.0955 | 0.0569 | 5208 |
| Upper-to-lower differential | sex: female vs male | 0.2588 | 0.0341 | <0.001 | 0.1920 | 0.3256 | 5208 |
| Upper-to-lower differential | Constitution: Qi-deficiency vs Balanced | 0.1542 | 0.0933 | 0.098 | -0.0287 | 0.3371 | 5208 |
| Upper-to-lower differential | Constitution: Yang-deficiency vs Balanced | 0.0583 | 0.0503 | 0.246 | -0.0402 | 0.1568 | 5208 |
| Upper-to-lower differential | Constitution: Yin-deficiency vs Balanced | 0.0855 | 0.0519 | 0.100 | -0.0163 | 0.1872 | 5208 |
| Upper-to-lower differential | Constitution: Phlegm-dampness vs Balanced | 0.0097 | 0.0448 | 0.829 | -0.0781 | 0.0974 | 5208 |
| Upper-to-lower differential | Constitution: Damp-heat vs Balanced | 0.0240 | 0.1294 | 0.853 | -0.2296 | 0.2776 | 5208 |
| Upper-to-lower differential | Constitution: Qi stagnation vs Balanced | 0.2132 | 0.1090 | 0.050 | -0.0004 | 0.4267 | 5208 |
| Upper-to-lower differential | Constitution: Blood stasis vs Balanced | -0.0673 | 0.1259 | 0.593 | -0.3141 | 0.1795 | 5208 |
| Upper-to-lower differential | Constitution: Special diathesis vs Balanced | 0.0735 | 0.1987 | 0.711 | -0.3160 | 0.4631 | 5208 |
| Upper-to-lower differential | Age | 0.0092 | 0.0028 | 0.001 | 0.0036 | 0.0147 | 5208 |
| Upper-to-lower differential | BMI | -0.0537 | 0.0058 | <0.001 | -0.0652 | -0.0423 | 5208 |
| Upper-to-lower differential | Abdominal skinfold thickness | -0.0023 | 0.0015 | 0.114 | -0.0052 | 0.0006 | 5208 |
| Upper-to-lower differential | Systolic blood pressure | 0.0032 | 0.0010 | 0.002 | 0.0012 | 0.0053 | 5208 |
| Upper-to-lower differential | Diastolic blood pressure | -0.0050 | 0.0016 | 0.001 | -0.0081 | -0.0019 | 5208 |
| Trunk-to-limb differential | Intercept | 1.5909 | 0.2035 | <0.001 | 1.1920 | 1.9898 | 5208 |
| Trunk-to-limb differential | broad_region: middle vs cold | 0.0009 | 0.0711 | 0.990 | -0.1386 | 0.1403 | 5208 |
| Trunk-to-limb differential | broad_region: warm vs cold | 0.0161 | 0.0308 | 0.602 | -0.0443 | 0.0765 | 5208 |
| Trunk-to-limb differential | sex: female vs male | 0.1367 | 0.0270 | <0.001 | 0.0838 | 0.1897 | 5208 |
| Trunk-to-limb differential | Constitution: Qi-deficiency vs Balanced | 0.1238 | 0.0740 | 0.094 | -0.0211 | 0.2688 | 5208 |
| Trunk-to-limb differential | Constitution: Yang-deficiency vs Balanced | 0.0690 | 0.0398 | 0.083 | -0.0090 | 0.1470 | 5208 |
| Trunk-to-limb differential | Constitution: Yin-deficiency vs Balanced | 0.1312 | 0.0411 | 0.001 | 0.0506 | 0.2118 | 5208 |
| Trunk-to-limb differential | Constitution: Phlegm-dampness vs Balanced | 0.0002 | 0.0355 | 0.996 | -0.0694 | 0.0697 | 5208 |
| Trunk-to-limb differential | Constitution: Damp-heat vs Balanced | -0.0216 | 0.1025 | 0.833 | -0.2224 | 0.1793 | 5208 |
| Trunk-to-limb differential | Constitution: Qi stagnation vs Balanced | 0.1814 | 0.0863 | 0.036 | 0.0122 | 0.3507 | 5208 |
| Trunk-to-limb differential | Constitution: Blood stasis vs Balanced | -0.1166 | 0.0997 | 0.242 | -0.3121 | 0.0788 | 5208 |
| Trunk-to-limb differential | Constitution: Special diathesis vs Balanced | 0.1542 | 0.1575 | 0.327 | -0.1544 | 0.4628 | 5208 |
| Trunk-to-limb differential | Age | 0.0101 | 0.0022 | <0.001 | 0.0057 | 0.0145 | 5208 |
| Trunk-to-limb differential | BMI | -0.0656 | 0.0046 | <0.001 | -0.0746 | -0.0565 | 5208 |
| Trunk-to-limb differential | Abdominal skinfold thickness | -0.0023 | 0.0011 | 0.041 | -0.0046 | -0.0001 | 5208 |
| Trunk-to-limb differential | Systolic blood pressure | 0.0040 | 0.0008 | <0.001 | 0.0023 | 0.0056 | 5208 |
| Trunk-to-limb differential | Diastolic blood pressure | -0.0027 | 0.0013 | 0.036 | -0.0053 | -0.0002 | 5208 |
| Anterior-to-posterior differential | Intercept | -0.1471 | 0.1345 | 0.274 | -0.4109 | 0.1167 | 5208 |
| Anterior-to-posterior differential | broad_region: middle vs cold | -0.1087 | 0.0467 | 0.020 | -0.2003 | -0.0171 | 5208 |
| Anterior-to-posterior differential | broad_region: warm vs cold | -0.1619 | 0.0203 | <0.001 | -0.2016 | -0.1222 | 5208 |
| Anterior-to-posterior differential | sex: female vs male | 0.0147 | 0.0177 | 0.406 | -0.0200 | 0.0495 | 5208 |
| Anterior-to-posterior differential | Constitution: Qi-deficiency vs Balanced | -0.0507 | 0.0486 | 0.296 | -0.1459 | 0.0445 | 5208 |
| Anterior-to-posterior differential | Constitution: Yang-deficiency vs Balanced | -0.0218 | 0.0261 | 0.404 | -0.0730 | 0.0294 | 5208 |
| Anterior-to-posterior differential | Constitution: Yin-deficiency vs Balanced | -0.0261 | 0.0270 | 0.333 | -0.0791 | 0.0268 | 5208 |
| Anterior-to-posterior differential | Constitution: Phlegm-dampness vs Balanced | 0.0576 | 0.0233 | 0.013 | 0.0119 | 0.1033 | 5208 |
| Anterior-to-posterior differential | Constitution: Damp-heat vs Balanced | -0.0315 | 0.0673 | 0.640 | -0.1634 | 0.1004 | 5208 |
| Anterior-to-posterior differential | Constitution: Qi stagnation vs Balanced | -0.0434 | 0.0567 | 0.443 | -0.1545 | 0.0676 | 5208 |
| Anterior-to-posterior differential | Constitution: Blood stasis vs Balanced | -0.1117 | 0.0655 | 0.088 | -0.2400 | 0.0167 | 5208 |
| Anterior-to-posterior differential | Constitution: Special diathesis vs Balanced | 0.0097 | 0.1034 | 0.925 | -0.1929 | 0.2124 | 5208 |
| Anterior-to-posterior differential | Age | 0.0011 | 0.0015 | 0.436 | -0.0017 | 0.0040 | 5208 |
| Anterior-to-posterior differential | BMI | 0.0154 | 0.0030 | <0.001 | 0.0095 | 0.0214 | 5208 |
| Anterior-to-posterior differential | Abdominal skinfold thickness | 0.0006 | 0.0008 | 0.448 | -0.0009 | 0.0021 | 5208 |
| Anterior-to-posterior differential | Systolic blood pressure | -0.0018 | 0.0005 | 0.001 | -0.0028 | -0.0007 | 5208 |
| Anterior-to-posterior differential | Diastolic blood pressure | 0.0009 | 0.0008 | 0.267 | -0.0007 | 0.0026 | 5208 |

| **Table S6. Relative importance of individual body composition variables within the body composition block for each thermal metric.** | | | | | | |
| --- | --- | --- | --- | --- | --- | --- |
| **Outcome** | **Variable** | **Relative contribution within body composition block (%)** | **SD** | **Contribution to total explained variance (%)** | **SD** | **Rank** |
| back_avg | BMI | 74.29 | 3.00 | 32.32 | 0.94 | 1 |
| back_avg | fbpz | 19.97 | 2.78 | 8.70 | 1.29 | 2 |
| back_avg | diastolic_bp | 4.67 | 1.15 | 2.03 | 0.52 | 3 |
| back_avg | systolic_bp | 1.07 | 0.34 | 0.47 | 0.14 | 4 |
| front_avg | BMI | 72.25 | 2.33 | 24.74 | 0.53 | 1 |
| front_avg | fbpz | 16.70 | 1.64 | 5.72 | 0.60 | 2 |
| front_avg | diastolic_bp | 9.33 | 1.50 | 3.20 | 0.55 | 3 |
| front_avg | systolic_bp | 1.72 | 0.38 | 0.59 | 0.13 | 4 |
| front_back_diff | BMI | 85.55 | 2.79 | 18.95 | 0.29 | 1 |
| front_back_diff | systolic_bp | 7.98 | 2.35 | 1.78 | 0.60 | 2 |
| front_back_diff | fbpz | 5.89 | 0.60 | 1.31 | 0.15 | 3 |
| front_back_diff | diastolic_bp | 0.59 | 0.18 | 0.13 | 0.04 | 4 |
| trunk_limb_diff | BMI | 49.14 | 4.97 | 12.86 | 0.72 | 1 |
| trunk_limb_diff | systolic_bp | 30.57 | 7.62 | 8.13 | 2.44 | 2 |
| trunk_limb_diff | diastolic_bp | 14.64 | 4.25 | 3.85 | 1.11 | 3 |
| trunk_limb_diff | fbpz | 5.66 | 5.96 | 1.44 | 1.44 | 4 |
| upper_lower_diff | diastolic_bp | 37.14 | 5.94 | 7.08 | 1.37 | 1 |
| upper_lower_diff | BMI | 28.62 | 4.77 | 5.38 | 0.56 | 2 |
| upper_lower_diff | systolic_bp | 27.81 | 11.04 | 5.49 | 2.60 | 3 |
| upper_lower_diff | fbpz | 6.43 | 6.09 | 1.12 | 0.88 | 4 |
| Relative contribution within the body composition block was estimated using relative importance analysis (LMG/Shapley decomposition) and sums to 100% within each outcome. Contribution to total explained variance was calculated by multiplying the block-level contribution by the relative importance of each variable within the block. Results are pooled across multiply imputed datasets. | | | | | | |

| **Table S7. Dominant constitution contributors to thermal phenotypes within the constitution block.** | | | |
| --- | --- | --- | --- |
| **Outcome** | **Constitution category** | **Contribution within constitution block (%)** | **Rank** |
| back_avg | Phlegm-dampness | 71.62 | 1 |
| back_avg | Yin-deficiency | 13.67 | 2 |
| back_avg | Yang-deficiency | 3.67 | 3 |
| back_avg | Qi stagnation | 3.64 | 4 |
| back_avg | Damp-heat | 3.57 | 5 |
| back_avg | Blood stasis | 2.64 | 6 |
| back_avg | Qi-deficiency | 1.02 | 7 |
| back_avg | Special diathesis | 0.17 | 8 |
| front_avg | Phlegm-dampness | 72.27 | 1 |
| front_avg | Damp-heat | 10.02 | 2 |
| front_avg | Yin-deficiency | 7.83 | 3 |
| front_avg | Qi stagnation | 4.30 | 4 |
| front_avg | Yang-deficiency | 3.24 | 5 |
| front_avg | Qi-deficiency | 0.96 | 6 |
| front_avg | Blood stasis | 0.84 | 7 |
| front_avg | Special diathesis | 0.54 | 8 |
| front_back_diff | Phlegm-dampness | 63.52 | 1 |
| front_back_diff | Yang-deficiency | 14.88 | 2 |
| front_back_diff | Blood stasis | 12.64 | 3 |
| front_back_diff | Yin-deficiency | 4.85 | 4 |
| front_back_diff | Damp-heat | 1.36 | 5 |
| front_back_diff | Qi stagnation | 1.19 | 6 |
| front_back_diff | Qi-deficiency | 1.15 | 7 |
| front_back_diff | Special diathesis | 0.41 | 8 |
| trunk_limb_diff | Yang-deficiency | 46.31 | 1 |
| trunk_limb_diff | Damp-heat | 14.31 | 2 |
| trunk_limb_diff | Blood stasis | 14.24 | 3 |
| trunk_limb_diff | Yin-deficiency | 9.66 | 4 |
| trunk_limb_diff | Phlegm-dampness | 9.01 | 5 |
| trunk_limb_diff | Qi stagnation | 4.94 | 6 |
| trunk_limb_diff | Special diathesis | 0.86 | 7 |
| trunk_limb_diff | Qi-deficiency | 0.67 | 8 |
| upper_lower_diff | Yang-deficiency | 57.07 | 1 |
| upper_lower_diff | Blood stasis | 16.53 | 2 |
| upper_lower_diff | Damp-heat | 12.75 | 3 |
| upper_lower_diff | Qi stagnation | 9.92 | 4 |
| upper_lower_diff | Phlegm-dampness | 2.13 | 5 |
| upper_lower_diff | Qi-deficiency | 0.80 | 6 |
| upper_lower_diff | Yin-deficiency | 0.76 | 7 |
| upper_lower_diff | Special diathesis | 0.03 | 8 |

**Constitution classification overlap and secondary profile analysis**

| **Table S8. Distribution of secondary constitution profiles according to predominant constitution categories** | | | | | |
| --- | --- | --- | --- | --- | --- |
| **Predominant constitution** | **Total participants (N)** | **Participants with at least one secondary constitution, n (%)** | **Participants without secondary constitution, n (%)** | **Mean number of secondary constitutions per participant (SD)** | **Most frequent secondary constitution category, n (%)** |
| Balanced | 1803 | 26 (1.4%) | 1,777 (98.6%) | 0.0 (0.2) | Qi-deficiency, 12 (0.7%) |
| Qi-deficiency | 168 | 91 (54.2%) | 77 (45.8%) | 0.8 (1.2) | Yin-deficiency, 28 (16.7%) |
| Yang-deficiency | 789 | 431 (54.6%) | 358 (45.4%) | 0.6 (0.7) | Yin-deficiency, 167 (21.2%) |
| Yin-deficiency | 712 | 335 (47.1%) | 377 (52.9%) | 0.5 (0.7) | Yang-deficiency, 123 (17.3%) |
| Phlegm-dampness | 1404 | 421 (30.0%) | 983 (70.0%) | 0.3 (0.5) | Yin-deficiency, 149 (10.6%) |
| Damp-heat | 84 | 43 (51.2%) | 41 (48.8%) | 0.6 (0.9) | Yin-deficiency, 14 (16.7%) |
| Blood stasis | 89 | 42 (47.2%) | 47 (52.8%) | 0.5 (0.6) | Phlegm-dampness, 10 (11.2%) |
| Qi stagnation | 122 | 70 (57.4%) | 52 (42.6%) | 0.7 (1.0) | Qi-deficiency, 22 (18.0%) |
| Special diathesis | 35 | 23 (65.7%) | 12 (34.3%) | 0.7 (0.7) | Yin-deficiency, 6 (17.1%) |
| Note. Secondary constitutions represent non-predominant constitution categories reported in the questionnaire. Predominant constitution was defined according to the highest constitution score. The Balanced constitution category included both “Balanced constitution” and “basic Balanced constitution” according to the questionnaire classification framework; therefore, a small number of participants within this combined category showed secondary constitution tendencies in overlap analyses. | | | | | |

| **Table S9. Pairwise overlap between predominant and secondary constitution categories** | | | | | | | | |
| --- | --- | --- | --- | --- | --- | --- | --- | --- |
| **Predominant constitution** | **Qi-deficiency** | **Yang-deficiency** | **Yin-deficiency** | **Phlegm-dampness** | **Damp-heat** | **Blood stasis** | **Qi stagnation** | **Special diathesis** |
| Balanced | 12 (0.7%) | 1 (0.1%) | 5 (0.3%) | 6 (0.3%) | 1 (0.1%) | 1 (0.1%) | 7 (0.4%) | 1 (0.1%) |
| Qi-deficiency | NA | 27 (16.1%) | 28 (16.7%) | 26 (15.5%) | 7 (4.2%) | 11 (6.5%) | 22 (13.1%) | 8 (4.8%) |
| Yang-deficiency | 56 (7.1%) | NA | 167 (21.2%) | 132 (16.7%) | 25 (3.2%) | 43 (5.4%) | 44 (5.6%) | 19 (2.4%) |
| Yin-deficiency | 32 (4.5%) | 123 (17.3%) | NA | 122 (17.1%) | 25 (3.5%) | 39 (5.5%) | 34 (4.8%) | 14 (2.0%) |
| Phlegm-dampness | 27 (1.9%) | 145 (10.3%) | 149 (10.6%) | NA | 55 (3.9%) | 35 (2.5%) | 22 (1.6%) | 12 (0.9%) |
| Damp-heat | 3 (3.6%) | 6 (7.1%) | 14 (16.7%) | 14 (16.7%) | NA | 7 (8.3%) | 3 (3.6%) | 4 (4.8%) |
| Blood stasis | 7 (7.9%) | 8 (9.0%) | 8 (9.0%) | 10 (11.2%) | 7 (7.9%) | NA | 3 (3.4%) | 3 (3.4%) |
| Qi stagnation | 22 (18.0%) | 17 (13.9%) | 20 (16.4%) | 17 (13.9%) | 4 (3.3%) | 8 (6.6%) | NA | 3 (2.5%) |
| Special diathesis | 2 (5.7%) | 3 (8.6%) | 6 (17.1%) | 6 (17.1%) | 5 (14.3%) | 1 (2.9%) | 3 (8.6%) | NA |
| Note. Secondary constitutions represent non-predominant constitution categories reported in the questionnaire. Predominant constitution was defined according to the highest constitution score. All constitution-tendency categories were excluded from these overlap analyses. | | | | | | | | |

**Sensitivity analyses and secondary analyses**

| **Table S10. Comparison of ΔT-based and absolute-temperature models for constitution–region associations** | | | | | | | | | |
| --- | --- | --- | --- | --- | --- | --- | --- | --- | --- |
| **Site** | **Constitution** | **β (ΔT model)** | **FDR p (ΔT)** | **Sig (ΔT)** | **β (Mean model)** | **FDR p (Mean)** | **Sig (Mean)** | **Direction concordant** | **Significant in mean model** |
| Epigastric | Qi-deficiency | 0.107 | 0.038 | * | 0.061 | 0.846 |  | Yes | No |
| Epigastric | Phlegm-dampness | -0.24 | <0.001 | *** | -0.25 | <0.001 | *** | Yes | Yes |
| Left hypochondrium | Yang-deficiency | -0.085 | <0.001 | *** | -0.043 | 0.846 |  | Yes | No |
| Left hypochondrium | Phlegm-dampness | -0.055 | 0.002 | ** | -0.06 | 0.579 |  | Yes | No |
| Left lower abdomen | Phlegm-dampness | 0.12 | <0.001 | *** | 0.116 | 0.065 |  | Yes | No |
| Left lumbar (posterior) | Yang-deficiency | 0.069 | 0.019 | * | 0.115 | 0.154 |  | Yes | No |
| Periumbilical (central abdomen) | Yang-deficiency | 0.103 | <0.001 | *** | 0.143 | 0.099 |  | Yes | No |
| Precordial (left anterior chest) | Yang-deficiency | -0.064 | 0.012 | * | -0.023 | 0.846 |  | Yes | No |
| Precordial (left anterior chest) | Phlegm-dampness | -0.058 | 0.004 | ** | -0.065 | 0.579 |  | Yes | No |
| Right chest | Phlegm-dampness | -0.081 | <0.001 | *** | -0.084 | 0.398 |  | Yes | No |
| Right chest | Special diathesis | 0.202 | 0.019 | * | 0.227 | 0.846 |  | Yes | No |
| Right lower abdomen | Phlegm-dampness | 0.154 | <0.001 | *** | 0.151 | 0.002 | ** | Yes | Yes |
| Right lumbar (posterior) | Yang-deficiency | 0.073 | 0.019 | * | 0.117 | 0.154 |  | Yes | No |
| Right lumbar (posterior) | Phlegm-dampness | 0.137 | <0.001 | *** | 0.077 | 0.400 |  | Yes | No |
| Suprapubic (lower mid-abdomen) | Yang-deficiency | -0.091 | 0.019 | * | -0.041 | 0.846 |  | Yes | No |
| Summary: N=15 ΔT-significant pairs; Concordant=15 (100.0%); Also significant in mean model=2 (13.3%). | | | | | | | | | |

| **Table S11. Overall constitution effects and post hoc constitution-specific comparisons for acupoints with significant overall constitution effects.** | | | | | | | | |
| --- | --- | --- | --- | --- | --- | --- | --- | --- |
| **acupoint** | **overall_wald_chi2** | **df** | **p_overall** | **n_min** | **n_max** | **p_overall_fdr** | **overall_sig** | **overall_significant** |
| Ciliao (BL32) | 25.832 | 8 | 0.001 | 5208 | 5208 | 0.024 | * | TRUE |
| Shangliao (BL31) | 22.791 | 8 | 0.004 | 5208 | 5208 | 0.038 | * | TRUE |
| Pishu (BL20) | 16.014 | 8 | 0.042 | 5208 | 5208 | 0.295 |  | FALSE |
| Weishu (BL21) | 14.574 | 8 | 0.068 | 5208 | 5208 | 0.357 |  | FALSE |
| Zhongwan (CV12) | 11.377 | 8 | 0.181 | 5208 | 5208 | 0.547 |  | FALSE |
| Guanyuan (CV4) | 11.922 | 8 | 0.155 | 5208 | 5208 | 0.547 |  | FALSE |
| Yaoyangguan (GV3) | 11.125 | 8 | 0.195 | 5208 | 5208 | 0.547 |  | FALSE |
| Qihaishu (BL24) | 10.884 | 8 | 0.208 | 5208 | 5208 | 0.547 |  | FALSE |
| Rugen (ST18) | 9.894 | 8 | 0.273 | 5208 | 5208 | 0.636 |  | FALSE |
| Zhongji (CV3) | 9.457 | 8 | 0.305 | 5208 | 5208 | 0.641 |  | FALSE |
| Qimen (LR14) | 8.570 | 8 | 0.380 | 5208 | 5208 | 0.672 |  | FALSE |
| Shenshu (BL23) | 7.443 | 8 | 0.490 | 5208 | 5208 | 0.672 |  | FALSE |
| Shenfeng (KI23) | 7.053 | 8 | 0.531 | 5208 | 5208 | 0.672 |  | FALSE |
| Shenque (CV8) | 7.739 | 8 | 0.459 | 5208 | 5208 | 0.672 |  | FALSE |
| Tianshu (ST25) | 6.930 | 8 | 0.544 | 5208 | 5208 | 0.672 |  | FALSE |
| Daju (ST27) | 7.567 | 8 | 0.477 | 5208 | 5208 | 0.672 |  | FALSE |
| Shuidao (ST28) | 7.985 | 8 | 0.435 | 5208 | 5208 | 0.672 |  | FALSE |
| Guilai (ST29) | 6.276 | 8 | 0.616 | 5208 | 5208 | 0.681 |  | FALSE |
| Zigong (EX-CA1) | 6.454 | 8 | 0.597 | 5208 | 5208 | 0.681 |  | FALSE |
| Mingmen (GV4) | 5.645 | 8 | 0.687 | 5208 | 5208 | 0.721 |  | FALSE |
| Danzhong (CV17) | 3.503 | 8 | 0.899 | 5208 | 5208 | 0.899 |  | FALSE |

| **Table S12. Sensitivity analysis comparing primary and alternative location-adjustment models using raw acquisition site.** | | | | | | | | |
| --- | --- | --- | --- | --- | --- | --- | --- | --- |
| Site | Constitution | β (sensitivity) | 95% CI | Raw p | FDR p | Direction consistent with primary | Significant in primary analysis | Significant in sensitivity analysis |
| Chest (upper thorax) | Qi-deficiency | 0.068 | [0.006, 0.131] | 0.031 | 0.134 | Yes | No | No |
| Chest (upper thorax) | Yang-deficiency | 0.040 | [0.006, 0.075] | 0.022 | 0.113 | Yes | No | No |
| Chest (upper thorax) | Yin-deficiency | 0.046 | [0.010, 0.082] | 0.012 | 0.072 | Yes | No | No |
| Chest (upper thorax) | Phlegm-dampness | 0.023 | [-0.006, 0.053] | 0.116 | 0.370 | Yes | No | No |
| Chest (upper thorax) | Damp-heat | -0.029 | [-0.126, 0.067] | 0.552 | 0.757 | Yes | No | No |
| Chest (upper thorax) | Qi stagnation | -0.025 | [-0.094, 0.044] | 0.475 | 0.737 | Yes | No | No |
| Chest (upper thorax) | Blood stasis | 0.051 | [-0.040, 0.143] | 0.270 | 0.575 | Yes | No | No |
| Chest (upper thorax) | Special diathesis | 0.051 | [-0.097, 0.200] | 0.499 | 0.737 | Yes | No | No |
| Right chest | Qi-deficiency | -0.023 | [-0.098, 0.051] | 0.538 | 0.752 | Yes | No | No |
| Right chest | Yang-deficiency | -0.039 | [-0.081, 0.004] | 0.073 | 0.268 | Yes | No | No |
| Right chest | Yin-deficiency | 0.036 | [-0.005, 0.077] | 0.082 | 0.293 | Yes | No | No |
| Right chest | Phlegm-dampness | -0.086 | [-0.121, -0.052] | <0.001 | <0.001 | Yes | Yes | Yes |
| Right chest | Damp-heat | -0.001 | [-0.111, 0.108] | 0.979 | 0.992 | No | No | No |
| Right chest | Qi stagnation | 0.020 | [-0.063, 0.102] | 0.641 | 0.809 | Yes | No | No |
| Right chest | Blood stasis | 0.074 | [-0.032, 0.181] | 0.171 | 0.468 | Yes | No | No |
| Right chest | Special diathesis | 0.209 | [0.078, 0.340] | 0.002 | 0.015 | Yes | Yes | Yes |
| Precordial (left anterior chest) | Qi-deficiency | -0.019 | [-0.093, 0.055] | 0.610 | 0.791 | Yes | No | No |
| Precordial (left anterior chest) | Yang-deficiency | -0.064 | [-0.103, -0.025] | 0.001 | 0.013 | Yes | Yes | Yes |
| Precordial (left anterior chest) | Yin-deficiency | 0.012 | [-0.027, 0.052] | 0.540 | 0.752 | Yes | No | No |
| Precordial (left anterior chest) | Phlegm-dampness | -0.063 | [-0.096, -0.031] | <0.001 | 0.001 | Yes | Yes | Yes |
| Precordial (left anterior chest) | Damp-heat | 0.002 | [-0.095, 0.099] | 0.965 | 0.992 | Yes | No | No |
| Precordial (left anterior chest) | Qi stagnation | 0.038 | [-0.039, 0.115] | 0.335 | 0.634 | Yes | No | No |
| Precordial (left anterior chest) | Blood stasis | 0.036 | [-0.067, 0.138] | 0.495 | 0.737 | Yes | No | No |
| Precordial (left anterior chest) | Special diathesis | 0.160 | [0.020, 0.300] | 0.025 | 0.115 | Yes | No | No |
| Right hypochondrium | Qi-deficiency | 0.000 | [-0.057, 0.058] | 0.990 | 0.992 | No | No | No |
| Right hypochondrium | Yang-deficiency | 0.005 | [-0.028, 0.038] | 0.768 | 0.910 | Yes | No | No |
| Right hypochondrium | Yin-deficiency | -0.039 | [-0.073, -0.006] | 0.022 | 0.113 | Yes | No | No |
| Right hypochondrium | Phlegm-dampness | -0.012 | [-0.038, 0.014] | 0.379 | 0.661 | Yes | No | No |
| Right hypochondrium | Damp-heat | 0.039 | [-0.039, 0.117] | 0.325 | 0.634 | Yes | No | No |
| Right hypochondrium | Qi stagnation | -0.004 | [-0.064, 0.056] | 0.892 | 0.962 | Yes | No | No |
| Right hypochondrium | Blood stasis | -0.028 | [-0.101, 0.046] | 0.458 | 0.737 | Yes | No | No |
| Right hypochondrium | Special diathesis | 0.051 | [-0.089, 0.190] | 0.476 | 0.737 | Yes | No | No |
| Left hypochondrium | Qi-deficiency | -0.025 | [-0.094, 0.043] | 0.469 | 0.737 | Yes | No | No |
| Left hypochondrium | Yang-deficiency | -0.085 | [-0.121, -0.048] | <0.001 | <0.001 | Yes | Yes | Yes |
| Left hypochondrium | Yin-deficiency | 0.005 | [-0.030, 0.041] | 0.760 | 0.910 | Yes | No | No |
| Left hypochondrium | Phlegm-dampness | -0.060 | [-0.090, -0.031] | <0.001 | <0.001 | Yes | Yes | Yes |
| Left hypochondrium | Damp-heat | 0.040 | [-0.054, 0.134] | 0.401 | 0.676 | Yes | No | No |
| Left hypochondrium | Qi stagnation | -0.047 | [-0.124, 0.030] | 0.232 | 0.521 | Yes | No | No |
| Left hypochondrium | Blood stasis | -0.025 | [-0.116, 0.067] | 0.600 | 0.789 | Yes | No | No |
| Left hypochondrium | Special diathesis | 0.012 | [-0.086, 0.111] | 0.809 | 0.945 | Yes | No | No |
| Right lower abdomen | Qi-deficiency | -0.048 | [-0.122, 0.026] | 0.203 | 0.493 | Yes | No | No |
| Right lower abdomen | Yang-deficiency | -0.014 | [-0.058, 0.029] | 0.515 | 0.749 | Yes | No | No |
| Right lower abdomen | Yin-deficiency | -0.042 | [-0.084, -0.000] | 0.049 | 0.187 | Yes | No | No |
| Right lower abdomen | Phlegm-dampness | 0.149 | [0.113, 0.185] | <0.001 | <0.001 | Yes | Yes | Yes |
| Right lower abdomen | Damp-heat | 0.062 | [-0.050, 0.174] | 0.280 | 0.575 | Yes | No | No |
| Right lower abdomen | Qi stagnation | -0.070 | [-0.173, 0.033] | 0.183 | 0.476 | Yes | No | No |
| Right lower abdomen | Blood stasis | -0.066 | [-0.174, 0.042] | 0.234 | 0.521 | Yes | No | No |
| Right lower abdomen | Special diathesis | -0.124 | [-0.246, -0.002] | 0.046 | 0.185 | Yes | No | No |
| Left lower abdomen | Qi-deficiency | -0.083 | [-0.152, -0.014] | 0.018 | 0.103 | Yes | No | No |
| Left lower abdomen | Yang-deficiency | -0.022 | [-0.072, 0.029] | 0.400 | 0.676 | Yes | No | No |
| Left lower abdomen | Yin-deficiency | 0.002 | [-0.039, 0.043] | 0.933 | 0.986 | No | No | No |
| Left lower abdomen | Phlegm-dampness | 0.115 | [0.076, 0.153] | <0.001 | <0.001 | Yes | Yes | Yes |
| Left lower abdomen | Damp-heat | 0.061 | [-0.034, 0.157] | 0.207 | 0.493 | Yes | No | No |
| Left lower abdomen | Qi stagnation | -0.087 | [-0.224, 0.049] | 0.210 | 0.493 | Yes | No | No |
| Left lower abdomen | Blood stasis | -0.061 | [-0.154, 0.033] | 0.203 | 0.493 | Yes | No | No |
| Left lower abdomen | Special diathesis | -0.160 | [-0.313, -0.008] | 0.039 | 0.163 | Yes | No | No |
| Epigastric | Qi-deficiency | 0.110 | [0.034, 0.186] | 0.004 | 0.028 | Yes | Yes | Yes |
| Epigastric | Yang-deficiency | 0.027 | [-0.021, 0.075] | 0.267 | 0.575 | Yes | No | No |
| Epigastric | Yin-deficiency | 0.014 | [-0.033, 0.061] | 0.573 | 0.774 | Yes | No | No |
| Epigastric | Phlegm-dampness | -0.245 | [-0.290, -0.201] | <0.001 | <0.001 | Yes | Yes | Yes |
| Epigastric | Damp-heat | -0.090 | [-0.213, 0.033] | 0.152 | 0.446 | Yes | No | No |
| Epigastric | Qi stagnation | 0.051 | [-0.048, 0.151] | 0.312 | 0.624 | Yes | No | No |
| Epigastric | Blood stasis | 0.058 | [-0.067, 0.182] | 0.362 | 0.644 | Yes | No | No |
| Epigastric | Special diathesis | 0.020 | [-0.195, 0.235] | 0.857 | 0.957 | Yes | No | No |
| Suprapubic (lower mid-abdomen) | Qi-deficiency | -0.047 | [-0.143, 0.049] | 0.337 | 0.634 | Yes | No | No |
| Suprapubic (lower mid-abdomen) | Yang-deficiency | -0.091 | [-0.150, -0.031] | 0.003 | 0.019 | Yes | Yes | Yes |
| Suprapubic (lower mid-abdomen) | Yin-deficiency | -0.038 | [-0.089, 0.014] | 0.153 | 0.446 | Yes | No | No |
| Suprapubic (lower mid-abdomen) | Phlegm-dampness | 0.054 | [0.007, 0.102] | 0.024 | 0.115 | Yes | No | No |
| Suprapubic (lower mid-abdomen) | Damp-heat | 0.115 | [-0.016, 0.245] | 0.086 | 0.293 | Yes | No | No |
| Suprapubic (lower mid-abdomen) | Qi stagnation | 0.001 | [-0.157, 0.158] | 0.992 | 0.992 | No | No | No |
| Suprapubic (lower mid-abdomen) | Blood stasis | -0.039 | [-0.183, 0.104] | 0.593 | 0.789 | Yes | No | No |
| Suprapubic (lower mid-abdomen) | Special diathesis | -0.142 | [-0.349, 0.064] | 0.176 | 0.468 | Yes | No | No |
| Periumbilical (central abdomen) | Qi-deficiency | 0.040 | [-0.033, 0.113] | 0.282 | 0.575 | Yes | No | No |
| Periumbilical (central abdomen) | Yang-deficiency | 0.103 | [0.056, 0.151] | <0.001 | <0.001 | Yes | Yes | Yes |
| Periumbilical (central abdomen) | Yin-deficiency | 0.030 | [-0.013, 0.074] | 0.173 | 0.468 | Yes | No | No |
| Periumbilical (central abdomen) | Phlegm-dampness | -0.019 | [-0.058, 0.021] | 0.352 | 0.644 | Yes | No | No |
| Periumbilical (central abdomen) | Damp-heat | -0.037 | [-0.141, 0.067] | 0.486 | 0.737 | Yes | No | No |
| Periumbilical (central abdomen) | Qi stagnation | 0.050 | [-0.057, 0.158] | 0.361 | 0.644 | Yes | No | No |
| Periumbilical (central abdomen) | Blood stasis | -0.004 | [-0.110, 0.101] | 0.939 | 0.986 | Yes | No | No |
| Periumbilical (central abdomen) | Special diathesis | -0.119 | [-0.277, 0.039] | 0.139 | 0.431 | Yes | No | No |
| Left lumbar (posterior) | Qi-deficiency | 0.019 | [-0.069, 0.106] | 0.675 | 0.841 | Yes | No | No |
| Left lumbar (posterior) | Yang-deficiency | 0.070 | [0.025, 0.114] | 0.002 | 0.017 | Yes | Yes | Yes |
| Left lumbar (posterior) | Yin-deficiency | 0.009 | [-0.035, 0.054] | 0.686 | 0.844 | Yes | No | No |
| Left lumbar (posterior) | Phlegm-dampness | 0.016 | [-0.023, 0.055] | 0.424 | 0.702 | Yes | No | No |
| Left lumbar (posterior) | Damp-heat | 0.013 | [-0.108, 0.135] | 0.827 | 0.945 | Yes | No | No |
| Left lumbar (posterior) | Qi stagnation | 0.022 | [-0.069, 0.112] | 0.639 | 0.809 | Yes | No | No |
| Left lumbar (posterior) | Blood stasis | 0.105 | [-0.021, 0.232] | 0.103 | 0.342 | Yes | No | No |
| Left lumbar (posterior) | Special diathesis | 0.021 | [-0.159, 0.200] | 0.823 | 0.945 | Yes | No | No |
| Right lumbar (posterior) | Qi-deficiency | -0.007 | [-0.096, 0.081] | 0.870 | 0.960 | Yes | No | No |
| Right lumbar (posterior) | Yang-deficiency | 0.073 | [0.026, 0.121] | 0.002 | 0.018 | Yes | Yes | Yes |
| Right lumbar (posterior) | Yin-deficiency | -0.003 | [-0.049, 0.043] | 0.891 | 0.962 | Yes | No | No |
| Right lumbar (posterior) | Phlegm-dampness | 0.132 | [0.091, 0.174] | <0.001 | <0.001 | Yes | Yes | Yes |
| Right lumbar (posterior) | Damp-heat | -0.004 | [-0.131, 0.122] | 0.945 | 0.986 | Yes | No | No |
| Right lumbar (posterior) | Qi stagnation | 0.032 | [-0.069, 0.132] | 0.537 | 0.752 | Yes | No | No |
| Right lumbar (posterior) | Blood stasis | -0.010 | [-0.120, 0.099] | 0.852 | 0.957 | Yes | No | No |
| Right lumbar (posterior) | Special diathesis | -0.026 | [-0.177, 0.126] | 0.740 | 0.899 | Yes | No | No |

| **Table S13. Sensitivity analysis using raw acquisition site as the location-adjustment variable** | | | | | | | | |
| --- | --- | --- | --- | --- | --- | --- | --- | --- |
| **Site** | **Constitution** | **β (sensitivity)** | **95% CI** | **Raw p** | **FDR p** | **Direction consistent with primary** | **Significant in primary analysis** | **Significant in sensitivity analysis** |
| Chest (upper thorax) | Qi-deficiency | 0.068 | [0.006, 0.131] | 0.031 | 0.134 | Yes | No | No |
| Chest (upper thorax) | Yang-deficiency | 0.040 | [0.006, 0.075] | 0.022 | 0.113 | Yes | No | No |
| Chest (upper thorax) | Yin-deficiency | 0.046 | [0.010, 0.082] | 0.012 | 0.072 | Yes | No | No |
| Chest (upper thorax) | Phlegm-dampness | 0.023 | [-0.006, 0.053] | 0.116 | 0.370 | Yes | No | No |
| Chest (upper thorax) | Damp-heat | -0.029 | [-0.126, 0.067] | 0.552 | 0.757 | Yes | No | No |
| Chest (upper thorax) | Qi stagnation | -0.025 | [-0.094, 0.044] | 0.475 | 0.737 | Yes | No | No |
| Chest (upper thorax) | Blood stasis | 0.051 | [-0.040, 0.143] | 0.270 | 0.575 | Yes | No | No |
| Chest (upper thorax) | Special diathesis | 0.051 | [-0.097, 0.200] | 0.499 | 0.737 | Yes | No | No |
| Right chest | Qi-deficiency | -0.023 | [-0.098, 0.051] | 0.538 | 0.752 | Yes | No | No |
| Right chest | Yang-deficiency | -0.039 | [-0.081, 0.004] | 0.073 | 0.268 | Yes | No | No |
| Right chest | Yin-deficiency | 0.036 | [-0.005, 0.077] | 0.082 | 0.293 | Yes | No | No |
| Right chest | Phlegm-dampness | -0.086 | [-0.121, -0.052] | <0.001 | <0.001 | Yes | Yes | Yes |
| Right chest | Damp-heat | -0.001 | [-0.111, 0.108] | 0.979 | 0.992 | No | No | No |
| Right chest | Qi stagnation | 0.020 | [-0.063, 0.102] | 0.641 | 0.809 | Yes | No | No |
| Right chest | Blood stasis | 0.074 | [-0.032, 0.181] | 0.171 | 0.468 | Yes | No | No |
| Right chest | Special diathesis | 0.209 | [0.078, 0.340] | 0.002 | 0.015 | Yes | Yes | Yes |
| Precordial (left anterior chest) | Qi-deficiency | -0.019 | [-0.093, 0.055] | 0.610 | 0.791 | Yes | No | No |
| Precordial (left anterior chest) | Yang-deficiency | -0.064 | [-0.103, -0.025] | 0.001 | 0.013 | Yes | Yes | Yes |
| Precordial (left anterior chest) | Yin-deficiency | 0.012 | [-0.027, 0.052] | 0.540 | 0.752 | Yes | No | No |
| Precordial (left anterior chest) | Phlegm-dampness | -0.063 | [-0.096, -0.031] | <0.001 | 0.001 | Yes | Yes | Yes |
| Precordial (left anterior chest) | Damp-heat | 0.002 | [-0.095, 0.099] | 0.965 | 0.992 | Yes | No | No |
| Precordial (left anterior chest) | Qi stagnation | 0.038 | [-0.039, 0.115] | 0.335 | 0.634 | Yes | No | No |
| Precordial (left anterior chest) | Blood stasis | 0.036 | [-0.067, 0.138] | 0.495 | 0.737 | Yes | No | No |
| Precordial (left anterior chest) | Special diathesis | 0.160 | [0.020, 0.300] | 0.025 | 0.115 | Yes | No | No |
| Right hypochondrium | Qi-deficiency | 0.000 | [-0.057, 0.058] | 0.990 | 0.992 | No | No | No |
| Right hypochondrium | Yang-deficiency | 0.005 | [-0.028, 0.038] | 0.768 | 0.910 | Yes | No | No |
| Right hypochondrium | Yin-deficiency | -0.039 | [-0.073, -0.006] | 0.022 | 0.113 | Yes | No | No |
| Right hypochondrium | Phlegm-dampness | -0.012 | [-0.038, 0.014] | 0.379 | 0.661 | Yes | No | No |
| Right hypochondrium | Damp-heat | 0.039 | [-0.039, 0.117] | 0.325 | 0.634 | Yes | No | No |
| Right hypochondrium | Qi stagnation | -0.004 | [-0.064, 0.056] | 0.892 | 0.962 | Yes | No | No |
| Right hypochondrium | Blood stasis | -0.028 | [-0.101, 0.046] | 0.458 | 0.737 | Yes | No | No |
| Right hypochondrium | Special diathesis | 0.051 | [-0.089, 0.190] | 0.476 | 0.737 | Yes | No | No |
| Left hypochondrium | Qi-deficiency | -0.025 | [-0.094, 0.043] | 0.469 | 0.737 | Yes | No | No |
| Left hypochondrium | Yang-deficiency | -0.085 | [-0.121, -0.048] | <0.001 | <0.001 | Yes | Yes | Yes |
| Left hypochondrium | Yin-deficiency | 0.005 | [-0.030, 0.041] | 0.760 | 0.910 | Yes | No | No |
| Left hypochondrium | Phlegm-dampness | -0.060 | [-0.090, -0.031] | <0.001 | <0.001 | Yes | Yes | Yes |
| Left hypochondrium | Damp-heat | 0.040 | [-0.054, 0.134] | 0.401 | 0.676 | Yes | No | No |
| Left hypochondrium | Qi stagnation | -0.047 | [-0.124, 0.030] | 0.232 | 0.521 | Yes | No | No |
| Left hypochondrium | Blood stasis | -0.025 | [-0.116, 0.067] | 0.600 | 0.789 | Yes | No | No |
| Left hypochondrium | Special diathesis | 0.012 | [-0.086, 0.111] | 0.809 | 0.945 | Yes | No | No |
| Right lower abdomen | Qi-deficiency | -0.048 | [-0.122, 0.026] | 0.203 | 0.493 | Yes | No | No |
| Right lower abdomen | Yang-deficiency | -0.014 | [-0.058, 0.029] | 0.515 | 0.749 | Yes | No | No |
| Right lower abdomen | Yin-deficiency | -0.042 | [-0.084, -0.000] | 0.049 | 0.187 | Yes | No | No |
| Right lower abdomen | Phlegm-dampness | 0.149 | [0.113, 0.185] | <0.001 | <0.001 | Yes | Yes | Yes |
| Right lower abdomen | Damp-heat | 0.062 | [-0.050, 0.174] | 0.280 | 0.575 | Yes | No | No |
| Right lower abdomen | Qi stagnation | -0.070 | [-0.173, 0.033] | 0.183 | 0.476 | Yes | No | No |
| Right lower abdomen | Blood stasis | -0.066 | [-0.174, 0.042] | 0.234 | 0.521 | Yes | No | No |
| Right lower abdomen | Special diathesis | -0.124 | [-0.246, -0.002] | 0.046 | 0.185 | Yes | No | No |
| Left lower abdomen | Qi-deficiency | -0.083 | [-0.152, -0.014] | 0.018 | 0.103 | Yes | No | No |
| Left lower abdomen | Yang-deficiency | -0.022 | [-0.072, 0.029] | 0.400 | 0.676 | Yes | No | No |
| Left lower abdomen | Yin-deficiency | 0.002 | [-0.039, 0.043] | 0.933 | 0.986 | No | No | No |
| Left lower abdomen | Phlegm-dampness | 0.115 | [0.076, 0.153] | <0.001 | <0.001 | Yes | Yes | Yes |
| Left lower abdomen | Damp-heat | 0.061 | [-0.034, 0.157] | 0.207 | 0.493 | Yes | No | No |
| Left lower abdomen | Qi stagnation | -0.087 | [-0.224, 0.049] | 0.210 | 0.493 | Yes | No | No |
| Left lower abdomen | Blood stasis | -0.061 | [-0.154, 0.033] | 0.203 | 0.493 | Yes | No | No |
| Left lower abdomen | Special diathesis | -0.160 | [-0.313, -0.008] | 0.039 | 0.163 | Yes | No | No |
| Epigastric | Qi-deficiency | 0.110 | [0.034, 0.186] | 0.004 | 0.028 | Yes | Yes | Yes |
| Epigastric | Yang-deficiency | 0.027 | [-0.021, 0.075] | 0.267 | 0.575 | Yes | No | No |
| Epigastric | Yin-deficiency | 0.014 | [-0.033, 0.061] | 0.573 | 0.774 | Yes | No | No |
| Epigastric | Phlegm-dampness | -0.245 | [-0.290, -0.201] | <0.001 | <0.001 | Yes | Yes | Yes |
| Epigastric | Damp-heat | -0.090 | [-0.213, 0.033] | 0.152 | 0.446 | Yes | No | No |
| Epigastric | Qi stagnation | 0.051 | [-0.048, 0.151] | 0.312 | 0.624 | Yes | No | No |
| Epigastric | Blood stasis | 0.058 | [-0.067, 0.182] | 0.362 | 0.644 | Yes | No | No |
| Epigastric | Special diathesis | 0.020 | [-0.195, 0.235] | 0.857 | 0.957 | Yes | No | No |
| Suprapubic (lower mid-abdomen) | Qi-deficiency | -0.047 | [-0.143, 0.049] | 0.337 | 0.634 | Yes | No | No |
| Suprapubic (lower mid-abdomen) | Yang-deficiency | -0.091 | [-0.150, -0.031] | 0.003 | 0.019 | Yes | Yes | Yes |
| Suprapubic (lower mid-abdomen) | Yin-deficiency | -0.038 | [-0.089, 0.014] | 0.153 | 0.446 | Yes | No | No |
| Suprapubic (lower mid-abdomen) | Phlegm-dampness | 0.054 | [0.007, 0.102] | 0.024 | 0.115 | Yes | No | No |
| Suprapubic (lower mid-abdomen) | Damp-heat | 0.115 | [-0.016, 0.245] | 0.086 | 0.293 | Yes | No | No |
| Suprapubic (lower mid-abdomen) | Qi stagnation | 0.001 | [-0.157, 0.158] | 0.992 | 0.992 | No | No | No |
| Suprapubic (lower mid-abdomen) | Blood stasis | -0.039 | [-0.183, 0.104] | 0.593 | 0.789 | Yes | No | No |
| Suprapubic (lower mid-abdomen) | Special diathesis | -0.142 | [-0.349, 0.064] | 0.176 | 0.468 | Yes | No | No |
| Periumbilical (central abdomen) | Qi-deficiency | 0.040 | [-0.033, 0.113] | 0.282 | 0.575 | Yes | No | No |
| Periumbilical (central abdomen) | Yang-deficiency | 0.103 | [0.056, 0.151] | <0.001 | <0.001 | Yes | Yes | Yes |
| Periumbilical (central abdomen) | Yin-deficiency | 0.030 | [-0.013, 0.074] | 0.173 | 0.468 | Yes | No | No |
| Periumbilical (central abdomen) | Phlegm-dampness | -0.019 | [-0.058, 0.021] | 0.352 | 0.644 | Yes | No | No |
| Periumbilical (central abdomen) | Damp-heat | -0.037 | [-0.141, 0.067] | 0.486 | 0.737 | Yes | No | No |
| Periumbilical (central abdomen) | Qi stagnation | 0.050 | [-0.057, 0.158] | 0.361 | 0.644 | Yes | No | No |
| Periumbilical (central abdomen) | Blood stasis | -0.004 | [-0.110, 0.101] | 0.939 | 0.986 | Yes | No | No |
| Periumbilical (central abdomen) | Special diathesis | -0.119 | [-0.277, 0.039] | 0.139 | 0.431 | Yes | No | No |
| Left lumbar (posterior) | Qi-deficiency | 0.019 | [-0.069, 0.106] | 0.675 | 0.841 | Yes | No | No |
| Left lumbar (posterior) | Yang-deficiency | 0.070 | [0.025, 0.114] | 0.002 | 0.017 | Yes | Yes | Yes |
| Left lumbar (posterior) | Yin-deficiency | 0.009 | [-0.035, 0.054] | 0.686 | 0.844 | Yes | No | No |
| Left lumbar (posterior) | Phlegm-dampness | 0.016 | [-0.023, 0.055] | 0.424 | 0.702 | Yes | No | No |
| Left lumbar (posterior) | Damp-heat | 0.013 | [-0.108, 0.135] | 0.827 | 0.945 | Yes | No | No |
| Left lumbar (posterior) | Qi stagnation | 0.022 | [-0.069, 0.112] | 0.639 | 0.809 | Yes | No | No |
| Left lumbar (posterior) | Blood stasis | 0.105 | [-0.021, 0.232] | 0.103 | 0.342 | Yes | No | No |
| Left lumbar (posterior) | Special diathesis | 0.021 | [-0.159, 0.200] | 0.823 | 0.945 | Yes | No | No |
| Right lumbar (posterior) | Qi-deficiency | -0.007 | [-0.096, 0.081] | 0.870 | 0.960 | Yes | No | No |
| Right lumbar (posterior) | Yang-deficiency | 0.073 | [0.026, 0.121] | 0.002 | 0.018 | Yes | Yes | Yes |
| Right lumbar (posterior) | Yin-deficiency | -0.003 | [-0.049, 0.043] | 0.891 | 0.962 | Yes | No | No |
| Right lumbar (posterior) | Phlegm-dampness | 0.132 | [0.091, 0.174] | <0.001 | <0.001 | Yes | Yes | Yes |
| Right lumbar (posterior) | Damp-heat | -0.004 | [-0.131, 0.122] | 0.945 | 0.986 | Yes | No | No |
| Right lumbar (posterior) | Qi stagnation | 0.032 | [-0.069, 0.132] | 0.537 | 0.752 | Yes | No | No |
| Right lumbar (posterior) | Blood stasis | -0.010 | [-0.120, 0.099] | 0.852 | 0.957 | Yes | No | No |
| Right lumbar (posterior) | Special diathesis | -0.026 | [-0.177, 0.126] | 0.740 | 0.899 | Yes | No | No |

**Figure S1. Overview of participant characteristics by region and sex.**

**Figure S2. Residual diagnostic plots for representative primary regression models based on the first imputed dataset.**

Residual diagnostics for representative regression models from the primary analyses are shown using the first imputed dataset. Left panels show fitted-versus-residual plots; right panels show normal Q–Q plots. Residual plots showed no major systematic patterns or marked heteroscedasticity, although mild departures from normality were present in the tails of some ΔT and differential-temperature models. Similar diagnostic patterns were observed across the remaining imputed datasets.
